# New mitochondrial genomes of parasites belonging to the *Leucocytozoon toddi* and *Haemoproteus nisi* groups (Haemosporida, Apicomplexa)

**DOI:** 10.1186/s13071-026-07244-0

**Published:** 2026-01-20

**Authors:** Josef Harl, Tanja Himmel, M. Andreína Pacheco, Herbert Weissenböck

**Affiliations:** 1 Department of Pathology, Medical University Vienna, Waehringer Guertel 18-20, 1090 Vienna, Austria; 2 Institute of Pathology, Department for Biological Sciences and Pathobiology, University of Veterinary Medicine Vienna, Veterinaerplatz 1, 1210 Vienna, Austria; 3 Biology Department/Institute of Genomics and Evolutionary Medicine (iGEM), Temple University, Philadelphia, PA 19122-1801 USA

**Keywords:** Haemosporida, Birds, Accipitriformes, New lineages, *Leucocytozoon toddi*, *Haemoproteus nisi*

## Abstract

**Background:**

Avian haemosporidians are single-celled eukaryotic parasites of vertebrates that require dipteran vectors for transmission. The genera *Plasmodium*, *Haemoproteus* and *Leucocytozoon* currently comprise over 5000 parasite lineages based on a 478-bp section of the mitochondrial cytochrome* b* gene, which is the standard DNA barcode for avian haemosporidians. The mitochondrial genomes of apicomplexan parasites are highly condensed, with a length of approximately 6000 bp, containing three coding genes (cytochrome* c* oxidase subunit I, cytochrome* c* oxidase subunit III and cytochrome* b*) and dispersed fragments of the small and large ribosomal RNA (rRNA) genes. Since the mitochondrial genomes are relatively conserved, they are valuable markers for studying the phylogenetic relationships between haemosporidian parasites. However, until recently, mitochondrial genomes were unavailable for parasites of the *Haemoproteus nisi* and *Leucocytozoon toddi* species groups, which are exclusive to accipitriform raptors and strongly diverged from other *Haemoproteus* and *Leucocytozoon* parasites.

**Methods:**

We screened 171 accipitriform raptors from Austria and Germany using new primers targeting the cytochrome* b* gene of a previously neglected *L. toddi* clade. We also developed a new primer assay that enables the amplification and sequencing of the complete mitochondrial genomes of haemosporidian parasites. This process involved long-range PCRs with lineage-specific primers placed within the cytochrome* b* gene, followed by five nested PCRs targeting conserved sequence regions.

**Results:**

Screening the accipitriform raptors revealed 10 new *L. toddi* group lineages. We sequenced 18 mitochondrial genomes belonging to five *H. nisi* group, nine *L. toddi* group, and two other *Leucocytozoon* lineages. Phylogenetic analyses based on mt genome sequences placed the *L. toddi* lineages within the genus *Leucocytozoon*, but the results did not support a monophyly of the genus *Haemoproteus*.

**Conclusions:**

The new nested PCR approach with lineage-specific primers used for the long-range PCRs described herein successfully enabled the sequencing of the complete mitochondrial genomes, even in samples with mixed infections. The mitochondrial genomes of the *H. nisi* and *L. toddi* group lineages are highly valuable for resolving the phylogenetic relationships of the order Haemosporida since these parasites belong to clades distinct from other *Haemoproteus* and *Leucocytozoon* parasites.

**Graphical Abstract:**

**Figure:**
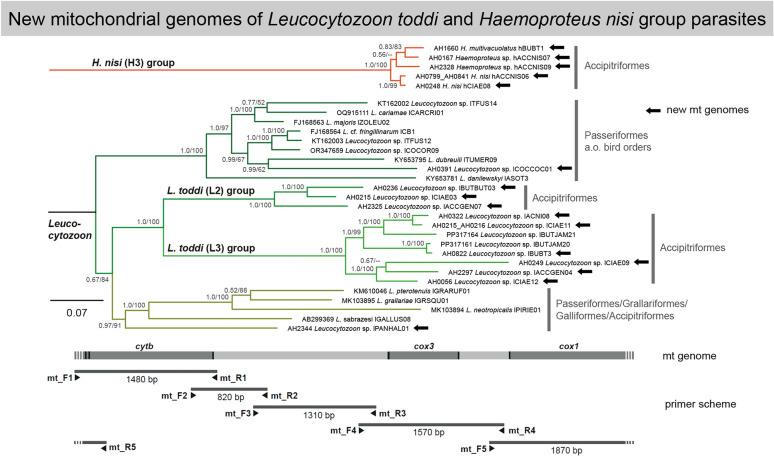

**Supplementary Information:**

The online version contains supplementary material available at 10.1186/s13071-026-07244-0.

## Background

Haemosporidian parasites (order Haemosporida, phylum Apicomplexa) are obligate heteroxenous blood and tissue parasites of vertebrates requiring blood-sucking dipteran vectors for transmission. The genera *Plasmodium* (family Plasmodiidae), *Haemoproteus* (family Haemoproteidae) and *Leucocytozoon* (family Leucocytozoidae) contain almost all known species of haemosporidian parasites from birds [1]. The MalAvi database [2], which includes a collection of avian haemosporidian parasite lineages based on a 478-bp “DNA barcode” section of the mitochondrial (mt) cytochrome* b* gene (*cytb*), currently lists over 5000 unique parasite lineages, with approximately 2000 *Haemoproteus*, 1600 *Plasmodium*, and 1600 *Leucocytozoon* lineages (last updated on 6 November 2025). Although highly valuable as a DNA barcode sequence, the short 478-bp *cytb* section does not provide sufficient information to resolve the phylogenetic relationships of the diverse haemosporidian parasite clades. Therefore, researchers attempted to estimate the phylogenetic relationships of haemosporidian parasites based on complete or nearly complete mt genomes [3–5]. The mt genomes of apicomplexan parasites are exceptional because they are highly condensed with a length of around 6000 bp, containing only three protein-coding genes (cytochrome* c* oxidase subunit I [*cox1*], cytochrome* c* oxidase subunit III [*cox3*] and *cytb*) and multiple dispersed fragments of the small and large ribosomal RNA (rRNA) genes [6, 7]. The gene order and general sequence patterns are conserved in most haemosporidian parasites, except for two *Nycteria* species that have highly rearranged mt genome structures [8].

The mt genome of the human malaria parasite *Plasmodium falciparum* was published more than 20 years ago [9]. Since then, mt genomes of human and simian *Plasmodium* species have been sequenced extensively for phylogenetic and population genetic studies [10–15]. The mt genomes of three avian haemosporidian parasites infecting domestic chickens were also sequenced almost two decades ago [16, 17]. Pacheco et al*.* [3] published the first comprehensive study on mt genomes of avian haemosporidian parasites, reporting 65 nearly complete *Plasmodium*, *Haemoproteus* and *Leucocytozoon* genomes. Additional mt genomes were published by Ciloglu et al*.* [18] and Musa [19]. However, these studies did not include parasite lineages of the *Leucocytozoon toddi* and *Haemoproteus nisi* groups, which are exclusive to accipitriform raptors and strongly diverged from their congenerics. Parasites of the two clades are among the most common pathogens in this host group, with prevalences > 25% [19–21], but they were overlooked in many studies because the primers of the common nested PCR protocol by Hellgren et al*.* [22] do not amplify the *cytb* of these lineages due to multiple mismatches in the primer sequences. However, using alternative primers, about 50 *L. toddi* group and six *H. nisi* group *cytb* lineages were identified in accipitriform raptors from Europe and North America [20, 21, 23–25]. The mt genomes of each two *L. toddi* group and *H. nisi* group lineages were only published in 2024 [21, 26].

The central aim of the current study was to obtain additional mt genomes of *L. toddi* and *H. nisi* group lineages, thereby providing better insights into their phylogenetic relationships. To this end, we designed primers specifically targeting a previously neglected subclade of *L. toddi* parasites, which were used to re-screen a set of 171 accipitriform raptors previously investigated by Harl et al*.* [20, 21]. Moreover, we developed a new PCR assay to amplify and sequence the complete mt genomes of haemosporidian parasites, even from samples containing mixed infections. In the present study, the method was used to sequence the mt genomes of several parasite lineages belonging to the *H. nisi* and *L. toddi* groups, as well as two other *Leucocytozoon* parasites.

## Methods

### *Cytb* screening of accipitriform raptors from Austria

To detect additional parasite lineages of the *L. toddi* group, we re-screened 171 accipitriform raptors from Austria with a new nested PCR assay using primers specifically targeting the second *L. toddi* group clade (referred to hereafter as the “L3 clade”). The sample included the following species and numbers of individuals: *Accipiter nisus* (22 individuals), *Aquila chrysaetos* (1), *Aquila heliaca* (10), *Astur gentilis* (9), *Buteo buteo* (70), *Buteo lagopus* (1), *Buteo* sp. (5), *Circus aeruginosus* (22), *Circus cyaneus* (4), *Clanga pomarina* (2), *Gypaetus barbatus* (2), *Gyps fulvus* (1), *Haliaeetus albicilla* (12), *Milvus milvus* (8), *Pandion haliaetus* (1) and *Pernis apivorus* (1). As described previously, blood samples were taken from 59 birds which were treated at the Service Unit for Birds and Reptiles of the Clinic for Small Animal Internal Medicine (Department for Companion Animals and Horses, VetMedUni Vienna) between 2015 and 2016, and tissue samples (liver and lung) were taken from 112 deceased birds which were received by the Research Institute of Wildlife Ecology (FIWI, Department of Interdisciplinary Life Sciences, VetMedUni Vienna) between 2009 and 2018 [20]. The birds mainly originated from Vienna, Lower Austria, and Burgenland (Additional file 1: Table S1). Species identification was performed by veterinarians at the two institutions. DNA was extracted either from tissue (liver and spleen) or blood spots using the DNeasy Blood & Tissue Kit (QIAGEN, Venlo, The Netherlands), following the manufacturer’s protocol for the isolation of DNA from tissue samples. The samples had been previously screened with the nested PCR assay by Hellgren et al. [22], which is a nested PCR assay targeting parasite lineages of one *L. toddi* group clade (referred to hereafter as the “L2 group”) [20], and a nested PCR assay targeting parasites of the *H. nisi* species group (referred to as “H3 group” in the following) [21]. The lineage lBUTBUT12, present in co-infection with lBUBT3 in sample AH1952, was obtained using the primers CytB_HPL_intF1 and CytB_HPL_intR1 [27]. These primers and PCR protocol were also used to obtain the partial *cytb* sequence of lineage lPANHAL01 from *Pandion haliaetus* (AH2344), which was not part of the sample of 171 raptors. The lineage lACCGEN07 was obtained from one *A. gentilis* (AH2325) from Germany using the primers targeting the *L. toddi* L2 clade (see below), and the lineage lACCGEN04 was obtained from another *A. gentilis* (AH2297) from Germany using the primers targeting the *L. toddi* L3 clade

The nested PCRs with the primers used by Hellgren et al*.* [22] and those targeting the *L. toddi* L2 clade were previously performed using the GoTaq® G2 Flexi DNA Polymerase kit (Promega, Madison, WI, USA) in 25-µl volumes containing 14.375 µl nuclease-free water, 5 µl 5 × Green GoTaq Flexi Buffer, 2 µl MgCl_2_ solution (25 mM), 0.5 µl nucleotide mix (10 mM), 0.125 µl GoTaq G2 Flexi DNA Polymerase (5 units/µl), 1 µl each of the forward and reverse primers (10 mM) and 1 µl of DNA template [20]. The nested PCRs targeting the *L. toddi* L3 and *H. nisi* H3 clades were performed using the KAPA2G Fast HotStart PCR kit (Sigma Aldrich, St. Louis, MO, USA) in 25-µl volumes containing 12.5 µl KAPPA2G polymerase mix, 8.5 µl nuclease-free water, 1 µl MgCl_2_ solution (25 mM), 1 µl each of the forward and reverse primers (10 mM) and 1 µl of DNA template. The PCR cycling regimen consisted of an initial denaturation for 2 min at 94 °C, followed by 25 cycles (first PCRs)/35 cycles (nested PCRs) of 30 s at 94 °C, 30 s at the respective annealing temperatures (see following sections) and 1 min at 72°, with a final extension at 72 °C for 10 min. Each 1-µl sample of the first PCR product was used as a template in the nested PCRs. The PCRs with the primers CytB_HPL_intF1 (5ʹ-GAG AAT TAT GGA GTG GAT GGT G-3ʹ) and CytB_HPL_intR1 (5ʹ-ATG TTT GCT TGG GAG CTG TAA TC-3ʹ) were performed with the GoTaq® Long PCR Master Mix (Promega) in 25-µl volumes containing 12.5 µl GoTaq® Long PCR Master Mix 2×, 9.5 µl nuclease-free water, 1 µl of each forward and reverse primer (10 mM) and 1 µl DNA template. The annealing temperature for the latter PCR was 55 °C, and the PCR conditions were the same as for the nested PCRs. Each 3 µl of PCR product was visualized in 1.5% agarose gels stained with ROTI®GelStain (Carl Roth, Karlsruhe, Germany), and the remaining PCR products were sent to Microsynth Austria (Vienna, Austria) for purification and Sanger sequencing in both directions using the PCR primers.

#### PCR assays with standard DNA barcoding primers

The 171 accipitriform raptors from Austria had been previously screened using the common nested PCR assay by Hellgren et al. [22], which allowed the amplification of partial *cytb* sequences (476/478 bp) of some parasite lineages [20]. The primers HaemNFI (5′-CAT ATA TTA AGA GAA NTA TGG AG-3′) and HaemNR3 (5′-ATA GAA AGAT AAG AAA TAC CAT TC-3′) were used in the first PCR. In the nested PCRs, the primers HaemF (5′-ATG GTG CTT TCG ATA TAT GCA TG-3′) and HaemR2 (5′-GCA TTA TCT GGA TGT GAT AAT GGT-3′) were used to amplify a 478-bp section in *Plasmodium* spp. and *Haemoproteus* spp., and theHaemFL (5′-ATG GTG TTT TAG ATA CTT ACA TT-3′) and HaemR2L (5′-CAT TAT CTG GAT GAG ATA ATG GIG C-3′) primers were used to amplify a 476-bp section in *Leucocytozoon* spp. The annealing temperatures were 50 °C in all PCR assays.

#### PCR assays targeting the* L. toddi* L2 clade

Partial *cytb* sequences (528 bp) of the first *L. toddi* group clade had been obtained previously with a nested PCR assay using the primers CytB_L2_F (5′-GAG AGT TAT GGG CTG GAT GGT-3′) and CytB_L2_R (5′-TAG AAA GCC AAG AAA TAC CAT TCT G-3′), and CytB_L2_nF (5′-GCT GGA TGG TGT TTT AGA TAY ATG C-3′) and CytB_L2_nR (5′-CCA TTC TGG AAC AAT ATG TAA AGG TG-3′). The first and the nested PCRs were both run at an annealing temperature of 54 °C [20]. The primers target most *L. toddi* L2 group lineages published until now, but they do not match sequences of parasite lineages of the *L. toddi* L3 group.

#### PCR assays targeting the* toddi * L3 clade

To date, the second *L. toddi* group clade was only represented by lineages lBUBT3 (EF607292), lBUTBUT12 (OL598531), lMILANS04 (JN164713), lBUTJAM20 (PP317158–62) and lBUTJAM21 (PP317163–65). To target parasites of this clade, we designed a new primer set to amplify the complete *cytb* in a nested PCR. The primers CytB_L3_F1 (5ʹ-ATG CCT AGA CGA ATT CCA GAT TAC TC-3′) and CytB_L3_R1 (5′-CAT GTC TTG CTA ACG ATT TGT ACG G-3′) amplify a 1460-bp fragment in the first PCR, and the primers CytB_L3_F2 (5′-CCA GAT TAC TCA GAC GAT TTA AAC GG-3′) and CytB_L3_R2 (5′-CCG CTC AAT GCT CAG AAA TGT C-3′) amplify a 1286-bp fragment (including the complete 1131 bp *cytb*) in the nested PCRs. The PCRs were run at annealing temperatures of 54 °C (first PCR) and 50 °C (nested PCR).

#### PCR assays targeting* Haemoproteus nisi* H3 clade

Partial *cytb* sequences (821 bp) of *H. nisi* group lineages were obtained previously from the 171 accipitriform raptors with a nested PCR using the primers CytB_Hnis_F1 (5ʹ-GGA GTA CTA CTT GCT ACC AGA T-3ʹ) and CytB_Hnis_R1 (5ʹ-GTT TGC TTG GG AGC TGT AAT C-3ʹ), and CytB_Hnis_F2 (5ʹ-TCA CCA GAA ATG GAT TAT GC-3ʹ) and CytB_Hnis_R2 (5ʹ-TGT GGT AAT GTA GAT CCT ATC C-3ʹ). The PCRs were run at annealing temperatures of 54 °C (first PCR) and 50 °C (nested PCR) [21].

### Sequencing and analysis of the mt genomes

#### Samples for mitochondrial genome analysis

The central aim of this study was to obtain additional mt genomes of parasites from the *L. toddi* and *H. nisi* groups. Most of the parasite lineages were selected from the *cytb* data sets obtained from 171 Austrian accipitriform raptors previously screened for haemosporidian parasites of the *L. toddi* L2 [28], *L. toddi* L3 (present study) and *H. nisi* H3 clades [21]. In addition, we included one *Pandion haliaetus* sent to the Institute of Pathology (VetMedUni Vienna, Austria) for routine diagnostics in 2018, and three *Astur gentilis* obtained from the State Laboratory Berlin-Brandenburg (Germany) in 2023. Apart from the accipitriform birds, we included one *Coccothraustes coccothraustes* infected with *Leucocytozoon* sp. lCOCCOC01 (Table 1). DNA was extracted from all samples using the DNeasy Blood & Tissue Kit (QIAGEN) following the manufacturer’s protocol for the isolation of DNA from tissue samples with slight modifications [20].

**Table 1:** Samples for mitochondrial genome analysis

ID	Host species	Parasite species	MalAvi lineage	Country	Material
*Leucocytozoon toddi L2 group*					
AH0215	*Circus aeruginosus*	*Leucocytozoon* sp.	lCIAE03	Austria	Liver
AH0236	*Buteo buteo*	*Leucocytozoon* sp.	lBUTBUT03	Austria	Liver
AH2325	*Astur gentilis*	*Leucocytozoon* sp.	lACCGEN07	Germany	Liver
*Leucocytozoon toddi L3 group*					
AH0056	*Circus aeruginosus*	*Leucocytozoon* sp.	lCIAE12	Austria	Liver
AH0215	*Circus aeruginosus*	*Leucocytozoon* sp.	lCIAE11	Austria	Liver
AH0216	*Circus aeruginosus*	*Leucocytozoon* sp.	lCIAE11	Austria	Liver
AH0249	*Circus aeruginosus*	*Leucocytozoon* sp.	lCIAE09	Austria	Liver
AH0322	*Accipiter nisus*	*Leucocytozoon* sp.	lACNI08	Austria	Liver
AH0822	*Buteo buteo*	*Leucocytozoon* sp.	lBUBT3	Austria	Blood spot
AH2297	*Astur gentilis*	*Leucocytozoon* sp.	lACCGEN04	Germany	LIver
*Leucocytozoon spp.*					
AH0391	*Coccothraustes coccothraustes*	*Leucocytozoon* sp.	lCOCCOC01	Austria	Liver
AH2344	*Pandion haliaetus*	*Leucocytozoon* sp.	lPANHAL01	Austria	Liver
*Haemoproteus nisi H3 group*					
AH0167	*Accipiter nisus*	*Haemoproteus* sp.	hACCNIS07	Austria	Blood spot
AH0248	*Circus aeruginosus*	*Haemoproteus nisi*	hCIAE08	Austria	Liver
AH0799	*Accipiter nisus*	*Haemoproteus nisi*	hACCNIS06	Austria	Blood spot
AH0841	*Accipiter nisus*	*Haemoproteus nisi*	hACCNIS06	Austria	Blood spot
AH1660	*Buteo buteo*	*Haemoproteus multivacuolatus*	hBUBT1	Austria	Blood spot
AH2328	*Astur gentilis*	*Haemoproteus* sp.	hACCNIS09	Germany	Liver

#### Mitochondrial genome primer assay

**Fig. 1:**
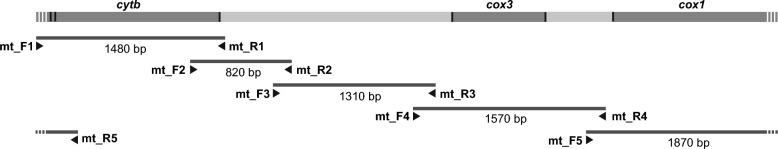
General primer scheme for sequencing the mitochondrial (mt) genomes of haemosporidian parasites. cox1, cox3, cytochrome* c* oxidase subunit 1 and 3 genes, respectively;* cytb*, cytochrome* b* gene

The primers for sequencing the complete mt genomes were designed based on an alignment containing a selection of complete (or nearly complete) mt genomes of haemosporidian parasites from birds, mammals and lizards (61 *Plasmodium*, 27 *Haemoproteus*, 21 *Leucocytozoon*, 2 *Nycteria* and 2 *Haemocystidium* genomes). The five universal primer sets (mt_F1/mt_R1 to mt_F5/mt_R5) were placed in conserved sequence regions and matched all mt genomes of haemosporidian parasites available in early 2024 when the primers were designed. The five resulting PCR fragments overlap each other and together cover the entire mt genome (Fig. 1; Table 2). The first mt genomes of the *L. toddi* and *H. nisi* species groups were published later in 2024 [21, 26]. Since the mt genomes of these two groups are strongly diverged from those of other *Haemoproteus* and *Leucocytozoon* parasites, alternative primers had to be designed for some PCR assays (Table 2). The primers of the first fragment (mt_F1/mt_R1) were placed in regions flanking the *cytb*, allowing the amplification of the entire gene. The main primers for the long-range PCR (mt_F2/mt_R5) were placed inside the *cytb* and allow amplification of the complete mt genome, excluding parts of the *cytb*. Since almost all samples analyzed in the present study contained mixed infections with multiple parasite lineages, we designed lineage-specific primers targeting the *cytb* of various parasite lineages (Table 3), which were used instead of the universal primers mt_F2/mt_R5 in the long-range PCRs. These primers were placed in regions close to the binding sites of the primers mt_F2/mt_R5, where the sequences of the targeted lineages differed sufficiently from other lineages contained in mixed infections. If the *cytb* sequences of the genomes of interest are known, this approach allows for amplifying and sequencing the mt genomes of multiple parasite lineages contained in a sample. Nonetheless, the electropherograms should be inspected for the presence of double peaks, which potentially indicate the presence of mixed infections. To avoid creating artificial chimeras, the electropherogram sections of the overlapping sequence regions must be checked carefully to ensure that they are identical.

**Table 2:** General primers for amplifying and sequencing the mitochondrial genomes of haemosporidian parasites

Primer	Sequence (5ʹ–3ʹ)	Tm^a^	Length of PCR product (bp)	Specificity
mt_F1	ATG CCT AGA CGT ATT CCT GAT TAT CC	60 °C	1480	All Haemosporida (except *L. toddi* group L3)
mt_R1	GCC AAC TCC CTG TCA TGT CTT	60 °C		All Haemosporida
mt_F2	TGA TTA CAG CTC CCA AGC AAA CA	61 °C	820	All Haemosporida
mt_R2	GTC AGG AAG TCC TGG ACG TTG	61 °C		All Haemosporida
mt_F3	GGA TTC TCT CCA CAC TTC AAT TCG TA	61 °C	1310	All Haemosporida (except *H. nisi* group H3)
mt_R3	ATG GCG AGA AGG GAA GTG TGT T	62 °C		All Haemosporida
mt_F4	CGC TAG TGT TTG CTT CTA ACA YTC C	60 °C	1570	All Haemosporida
mt_R4	ATA CAG TCC CAG CGA CAG CG	59 °C		All Haemosporida
mt_F5	CAT ACA AGA GAT CGC GTA CTT TGG	60 °C	1870	All Haemosporida
mt_R5	ACA CCA TCC ACT CCA TAA TTC TCT T	60 °C		All Haemosporida (except *L. toddi* group L3)
*Alternative PCR primers and primer combinations for Leucocytozoon toddi and Haemoproteus nisi group parasites*				
mt_F1_L3	ATG CCT AGA CGA ATT CCA GAT TAC TC	60 °C	1483	*L. toddi* L3 group (lACCGEN04, lACNI08, lBUBT3, lCIAE09, lCIAE11, lCIAE12)
mt_R1	GCC AAC TCC CTG TCA TGT CTT	60 °C		All Haemosporida
mt_F3_H3	GTT CCA ACA TTC TAG GTT TTT CGC G	61 °C	1265	*H. nisi* H3 group (hACCNIS06, hACCNIS07, hACCNIS09, hBUBT1, hCIAE08)
mt_R3	ATG GCG AGA AGG GAA GTG TGT T	62 °C		All Haemosporida

^a^Annealing temperature. Annealing temperatures were evaluated using Primer-BLAST implemented in the NCBI GenBank

**Table 3:** Lineage-specific primers placed inside the cytochrome* b* gene for targeted long-range PCR assays

ID	MalAvi lineage	Primers and primer sequences (5ʹ–3ʹ)
*Haemoproteus nisi H3 clade*		
AH0167	hACCNIS07	mt_F2_BUBT1: CTG AGT ATT GAG CGG AAC AAT ACA G; mt_R5_BUBT1: TCT GGT AGC AAG TAG TAC TCC AG
AH0248	hCIAE08	mt_F2_BUBT1: CTG AGT ATT GAG CGG AAC AAT ACA G; mt_R5_BUBT1: TCT GGT AGC AAG TAG TAC TCC AG
AH0799	hACCNIS06	mt_F2_BUBT1: CTG AGT ATT GAG CGG AAC AAT ACA G; mt_R5_BUBT1: TCT GGT AGC AAG TAG TAC TCC AG
AH1660	hBUBT1	mt_F2_BUBT1: CTG AGT ATT GAG CGG AAC AAT ACA G; mt_R5_BUBT1: TCT GGT AGC AAG TAG TAC TCC AG
AH0841	hACCNIS06	mt_F2_ACCNIS06: AGC TGG ATT AAT GAT TAT GAT GGG ATC; mt_R5_ACCNIS06: CAA ATG AAG CAC CAG TTG AAT G
AH2328	hACCNIS09	mt_F2_ACCNIS09: CAA TTA AAG TTA GTC TAT TTG TAA CTC CA; mt_R5_ACCNIS09: ACA AAT GAA GCA CCA GTT GC
*Leucocytozoon toddi L2 clade*		
AH0215	lCIAE03	mt_F2_CIAE03: CTG CGA TGA GAC GAC ATT TCT G; mt_R5_CIAE03: GTA TCT AAA ACA CCA TCC TGC CCA T
AH0236	lBUTBUT03	mt_F2_BUTBUT03: GCT CTA ATG TGT ATA GGA TGG CT; mt_R5_BUTBUT03: GCA TAT ATC TAA AAC ACC ATC CTG C
AH2325	lACCGEN07	mt_F2_ACCGEN07: GGA ATA TTA CCA TTG TCT CAT TCA G; mt_R5_ACCGEN07: AGG ATG CTC CAG CAG AGT
*Leucocytozoon toddi L3 clade*		
AH0056	lCIAE12	mt_F2_CIAE12: CTA TGC TTT AAT GTG CGT GGG ATA; mt_R5_CIAE12: TGC TAG TGA TGC TTC AGG AGT
AH0215	lCIAE11	mt_F2_CIAE11: CGA TGA GAC GAC ATT TCT GAG C; mt_R5_CIAE11: CAT GTC TTG CTA ACG ATT TGT ACG G
AH0216	lCIAE11	mt_F2_CIAE11: CGA TGA GAC GAC ATT TCT GAG C; mt_R5_CIAE11: CAT GTC TTG CTA ACG ATT TGT ACG G
AH0249	lCIAE09	mt_F2_CIAE09: GTG TAT AGG ATA TCA ATT ACC TAC ACT CG; mt_R5_CIAE09: TGC TAG TGA TGC TTC TGG AGT AT
AH0322	lACNI08	mt_F2_ACNI08: GTT CAT TCT ACG CTT TGA TGT G; mt_R5_ACNI08: ATG ATG CAC CAG TAG AAT GTA TG
AH0822	lBUBT3	mt_F2_BUBT3: GTT CAT TCT ATG CTT TAA TGT GCG T; mt_R5_BUBT3: CAA GAG ATG CTT CTG GAG TAT AGC A
AH2297	lACCGEN04	mt_F2_ACCGEN04: GTG GTG TAC TAG TAA TGT TAG CTG; mt_R5_ACCGEN04: ATA AAT GAT GCA CCA GTC GA
*Leucocytozoon spp.*		
AH0391	lCOCCOC01	mt_F2_COCCOC01_F2: GAT GCT AAA GCA TTT GCT TAC T; mt_R5_COCCOC01: ATA AAT ACA AAA GAT GCA CCT GTA G
AH2344	lPANHAL01	mt_F2: TGA TTA CAG CTC CCA AGC AAA CA; mt_R5_PANHAL01: GCG TTA AAT GTT TCT GGA GAA TAA CAA G

#### Long-range and nested PCRs

For the long-range PCRs, we used lineage-specific primers to target the parasite lineages of interest instead of the primers mt_F2 and mt_R5 (Table 3). The long-range PCRs were performed in 25-µl volumes using the GoTaq® Long PCR Master Mix (Promega). The Master Mix contained 12.5 µl GoTaq® Long PCR Master Mix 2×, 9.5 µl nuclease-free water, 1 µl each of forward and reverse primer (10 mM) and 1 µl DNA template. The PCR cycling regimen consisted of an initial denaturation for 2 min at 94 °C, followed by 35 cycles of 30 s at 94 °C, 30 s at 56 °C (annealing phase) and 5 min at 72 °C (extension phase), with a final extension for 10 min at 72 °C. Each 15-µl sample of the PCR products was visualized in 1.5% agarose gels stained with ROTI®GelStain (Carl Roth). The PCR products were excised from the gels using sterilized (flamed) spatulas, purified using the QIAquick Gel-Extraction Kit (QIAGEN) according to the manufacturer’s standard protocol and eluted with 20 µl nuclease-free water. The purified PCR products were then used as templates for the nested PCRs.

The nested PCRs were conducted with the primer sets mt_F2(or lineage-specific primers)/mt_R2, mt_F3/mt_R3, mt_F4/mt_R4, and mt_F5/mt_R5(or lineage-specific primers). In addition, PCRs were performed with the primer sets mt_F1/mt_R5(or lineage-specific primers) and mt_F5/mt_R1(or lineage-specific primers) to obtain the complete *cytb* and flanking regions in case the original *cytb* sequences were only partial. Alternative primers were used to amplify individual PCR fragments of the *L. toddi* L3 group (mt_F1_L3 instead of mt_F1) and *H. nisi* H3 group (mt_F3_H3 instead of mt_F3). The nested PCRs were all performed with the KAPA2G Fast HotStart PCR kit (Sigma Aldrich) in 25-µl reaction volumes containing 12.5 µl polymerase mix, 8.5 µl nuclease-free water, 1 µl MgCl₂ (25 mM), 1 µl each of forward and reverse primer (10 mM) and 1 µl of the purified PCR products from the long-range PCRs. The PCR cycling regimen consisted of an initial denaturation for 2 min at 95 °C, followed by 35 cycles of 30 s at 95 °C, 30 s at 56 °C (annealing phase), and 1 min at 72 °C (extension phase), with a final extension for 10 min at 72 °C. Each 3-µl sample of the PCR products was visualized in 1.5% agarose gels, and the remaining PCR products were sent to Microsynth Austria (Vienna, Austria) for purification and Sanger sequencing in both directions using the PCR primers.

**Fig. 2:**
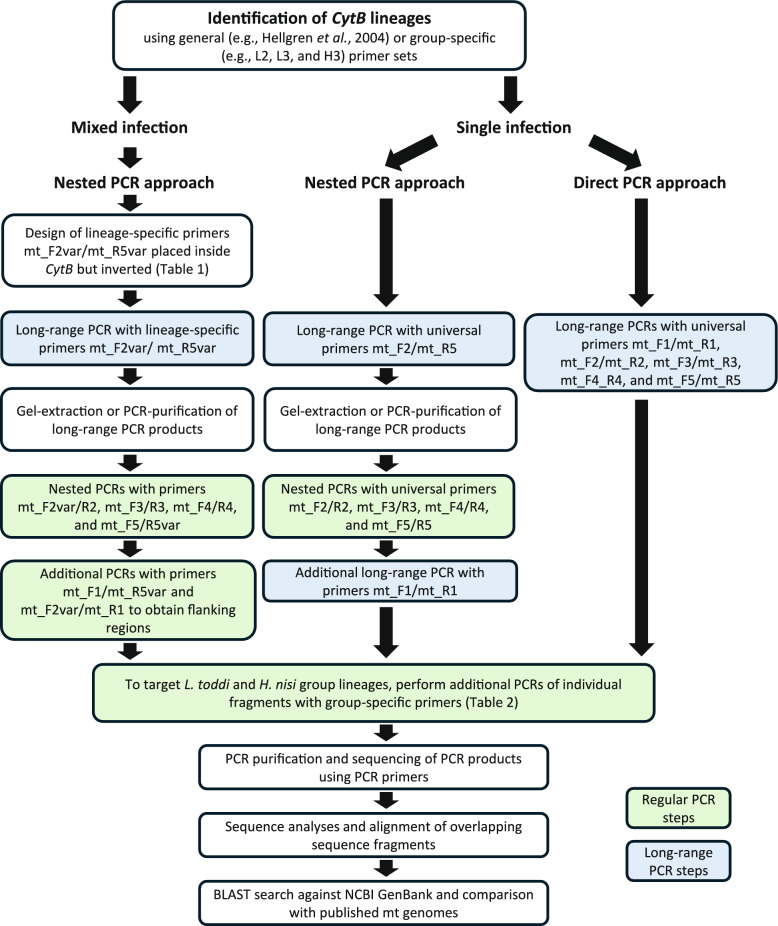
Flowchart depicting the process of sequencing the mitochondrial (mt) genomes of haemosporidian parasites from samples with mixed and single infections. * cytb*, cytochrome* b* gene

A flow chart depicting the workflow for sequencing the mt genomes in the present study and potential applications when working with samples containing single infections is provided in Fig. 2.

#### Sequence analyses

All raw forward and reverse sequences were aligned and visually inspected using BioEdit v. 7.7.1 [29]. The fragments of the complete mt genomes were also combined with BioEdit v. 7.7.1 after the primer ends were removed. Genetic distances, nucleotide compositions and amino acid compositions were evaluated with MEGA-X v. 10.0.5 [30].

#### Phylogenetic analysis of haemosporidian mt genomes

Phylogenetic trees were calculated based on an alignment including the three protein-coding genes (*cox1*, *co*x3, *cytb*) from a selection of 104 mt genomes of haemosporidian parasites, including the 18 mt genomes of 16 parasite lineages generated for the present study. The selection covered almost the entire diversity of available mt genomes from haemosporidian parasites. One mt genome per each species was selected for the mammalian haemosporidians. Regarding the avian *Haemoproteus*, *Plasmodium*, and *Leucocytozoon* lineages, the mt genomes of some parasite lineages that resembled others contained in the alignment were excluded to reduce the number of taxa. A list containing all mt genomes, GenBank accession numbers, alignments and information on host species and MalAvi lineages is provided in Additional file 2: Table S2. The mt genomes were aligned with MAFFT v7.311 [31] using the option “G-INS-I”, and the three coding genes were separated. The alignment for the phylogenetic calculations included 3315 bp in three partitions (*cox1*: 1431 bp; *cox3*: 753 bp; *cytb*: 1131 bp). Model tests were performed with IQ-TREE v. 3.0.1 [32], with the results suggesting GTR + F + I + G4 as the best-fit model for all three partitions according to the corrected Akaike Information Criterion (cAIC). A Bayesian Inference (BI) tree was calculated with MrBayes v. 3.2 [33] by running the analysis for 5 million generations and sampling every thousandth tree. After the first 25% of the trees were discarded as burn-in, a majority rule consensus tree was calculated from the remaining 3750 trees. A maximum likelihood (ML) majority rule consensus tree was calculated for the partitioned data set using IQ-TREE v. 3.0.1 [32] by performing 10,000 bootstrap replicates. In addition, we calculated phylogenetic trees using the same set of mt genomes but including non-coding sequence parts (excluding all gap positions) in the alignment. The best-fit model for the non-coding sequence parts according to the cAIC was also GTR + F + I + G4. The final alignment included 5484 bp (*cox1*: 1431 bp; *cox3*: 753 bp; *cytb*: 1131 bp; non-coding: 2169 bp). The BI and ML trees were calculated using the same programs and settings as for the alignment that included only the three protein-coding genes. The trees were visualized using FigTree v. 1.4.3 (http://tree.bio.ed.ac.uk/; Andrew Rambaut) and graphically edited with Adobe Illustrator CC v. 2015 (Adobe Inc., San Jose, CA, USA).

### Phylogenetic analysis of *Leucocytozoon toddi* group lineages

A phylogenetic tree was also calculated with all *cytb* lineages of the *L. toddi* group covering the entire 478-bp barcode region. The alignment included 60 different *L. toddi* group lineages, 46 of which belong to the L2 clade and 14 to the L3 clade. A sequence of *Leucocytozoon californicus* lCIAE02 (EF607287) was included as an outgroup. A model test was performed using IQ-TREE v. 3.0.1 [32], with the results suggesting TIM2 + F + G4 as the best-fit model according to the cAIC. However, since the latter model was not available in MrBayes v. 3.2 [33], the second-best model GTR + F + G4 was used for both the ML and BI analyses. A ML majority rule consensus tree was calculated with IQ-TREE v. 3.0.1 [32] by performing 10,000 bootstrap replicates. A BI tree was calculated with MrBayes v. 3.2 [33], running the analysis for 5 million generations and sampling every thousandth tree. After the first 25% of the trees were discarded as burn-in, a majority rule consensus tree was calculated from the remaining 3750 trees. The tree was visualized with FigTree v. 1.4.3 and graphically edited with Adobe Illustrator CC v. 2015 (Adobe Inc.).

## Results

### *Cytb* screening of accipitriform raptors from Austria

Using group-specific primers targeting the *L. toddi* L3 group, we obtained the complete *cytb* genes of 10 parasite lineages belonging to this clade (lACCGEN05, lACNI08, lBUBT3, lBUTBUT19, lBUTBUT20, lCIAE09, lCIAE10, lCIAE11, lCIAE12 and lMILANS04), of which only lBUBT3 and lMILANS04 were known previously [20, 25].

Combining the data from Harl et al*.* [20], Harl et al*.* [21] and the present study, 57.9% (99/171 individuals) of the accipitriform raptors were positive for haemosporidian parasites, whereby 15.8% (27 individuals [ind.]), 14.0% (24 ind.) and 4.1% (7 ind.) featured double, triple and quadruple infections, respectively. The prevalence of parasites of the *L. toddi* group (47.4%, 81 ind.) and of the *H. nisi* group (24.6%, 42 ind.) was particularly high, while other *Leucocytozoon*, *Haemoproteus*, and *Plasmodium* parasites were only rarely identified, with 5.9% (10 ind.), 1.2% (2 ind.) and 8.8% (15 ind.) positive individuals, respectively. Among the 81 raptors containing *L. toddi* group parasites, 57 and 49 individuals were infected with parasites of the *L. toddi* L2 and *L. toddi* L3 clades, respectively. Combining the data from the present study with data from the two previous studies of Harl et al. [20, 21], we recorded 50 different parasite lineages in the 171 accipitriform raptors; these belonged to the genus *Plasmodium* (6 lineages), *L. toddi* L2 group (21), *L. toddi* L3 group (11), other *Leucocytozoon* parasites (4), *H. nisi* H3 group (6) and subgenus *Parahaemoproteus* (2). A detailed list of all parasite lineages is provided in Additional file 1: Table S1. The other samples, which were not from the 171 raptors from Austria, also contained new parasite lineages. One *Astur gentilis* from Germany (AH2297) was infected with the lineage lACCGEN04 belonging to the *L. toddi* L3 clade, and another *A. gentilis* from Germany (AH2325) contained the lineage lACCGEN07 belonging to the *L. toddi* L2 clade. Moreover, one *P. haliaetus* from Austria (AH2344) was infected with the new *Leucocytozoon* lineage lPANHAL01, which also strongly diverged from other known *Leucocytozoon* lineages but was not part of the *L. toddi* group.

### New mt genomes

The complete mt genomes of 16 different parasite lineages were sequenced, including five *H. nisi* H3 group lineages (hACCNIS06, hACCNIS07, hACCNIS09, hBUBT1 and hCIAE08), three *L. toddi* L2 clade lineages (lACCGEN07, lBUTBUT03 and lCIAE03), six *L. toddi* L3 clade lineages (lACCGEN04, lACNI08, lBUBT3, lCIAE09, lCIAE11 and lCIAE12) and two other *Leucocytozoon* lineages (lCOCCOC01 and lPANHAL01). The new mt genomes are highly relevant because the parasites belong to phylogenetically distinct clades, which are strongly diverged from their congenerics, are exclusive to accipitriform raptors and are still poorly investigated in terms of their biology. The data allow for a better assessment of the phylogenetic relationships of members of Haemosporida and provide a basis for future studies investigating haemosporidian parasites of accipitriform raptors. The general organization of the 16 new mt genomes was identical to that of most other haemosporidian parasites. The mean G/C-contents for the *L. toddi* L2 group, the *L. toddi* L3 group, the other two *Leucocytozoon* lineages and the *H. nisi* H3 group were 32.4%, 29.2%, 30.0% and 25.5% (*cox1*); 27.1%, 21.2%, 26.6% and 21.4% (*cox3*); and 29.0%, 27.0%, 28.2%, and 22.6% (*cytb*). The mean G/C contents were generally lower in the *H. nisi* H3 group than in the *Leucocytozoon* lineages. Interestingly, the G/C-content differed strongly in the *cox3* of the *L. toddi* L2 group (27.1%) and *L. toddi* L3 group (21.2%) but was similar in the other two coding genes. Tables showing the nucleotide and amino acid compositions of the three protein-coding genes are provided in Additional file 3: Tables S3–S8. The "mold, protozoan and coelenterate" mitochondria code is used as the standard mt code for haemosporidian parasites. Accordingly, there are eight start codons (“TTA,” “TTG,” “CTG,” “ATT,” “ATC,” “ATA,” “ATG” and “GTG”) and two stop codons (“TAA” and “TAG”). However, start and stop codons could not be identified in the coding genes of some of the newly obtained and previously published mt genomes, indicating the use of alternative codons. More information relating to the nucleotide composition of the three protein-coding genes and codon usage is provided in Additional file 4: Text S1.

### Phylogeny of haemosporidian mt genomes

**Fig. 3:**
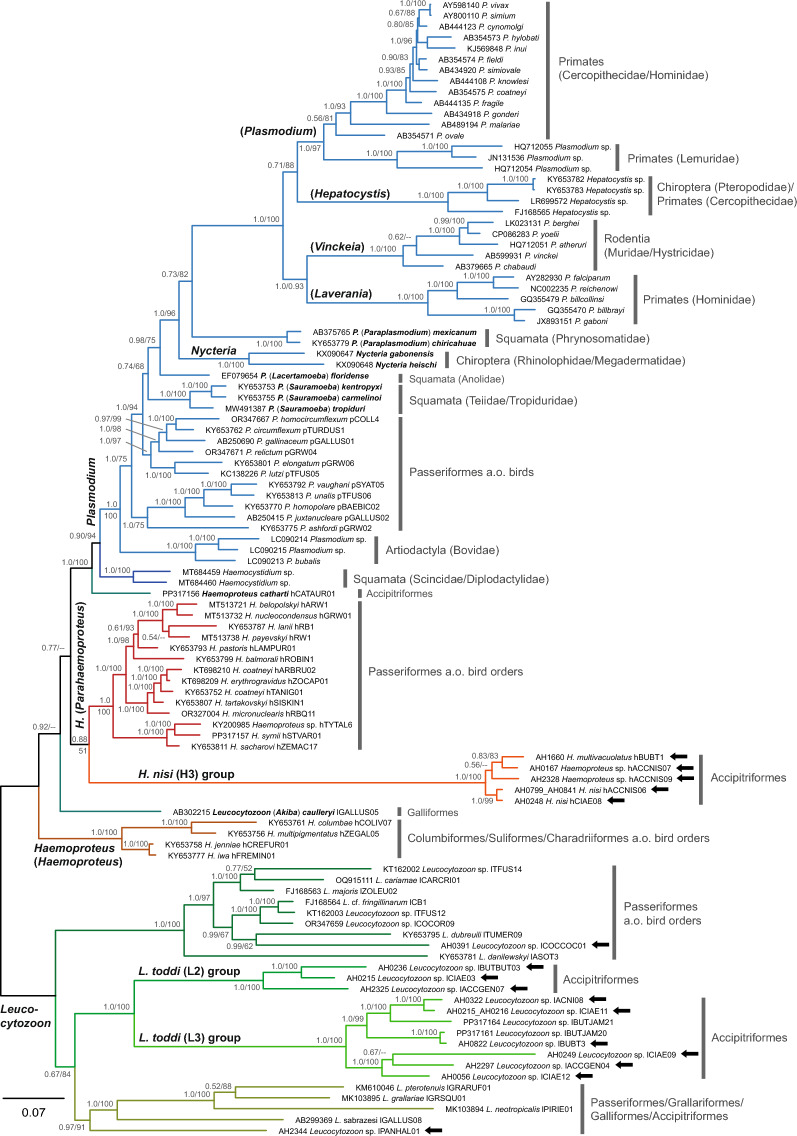
Phylogenetic relationships between haemosporidian parasite lineages based on the concatenated sequences of the three mitochondrial protein-coding genes *cox1* (1431 bp), *cox3* (753 bp) and *cytb* (1131 bp). Bayesian Inference posterior probabilities and maximum likelihood bootstrap values are indicated at all nodes. Bold black arrows mark the lineages of which the mitochondrial genomes were sequenced for the present study. For haemosporidian parasites of bird hosts, the name of the MalAvi lineage is included in the label. The main host groups are indicated to the right of the clades. a.o., And others;* cox1, cox3*, cytochrome* c* oxidase subunit 1 and 3 genes, respectively;* cytb*, cytochrome* b* gene

Phylogenetic BI and ML trees were calculated based on an alignment containing the three protein-coding genes (*cox1*, *cox3* and *cytb*) of 104 haemosporidian parasite lineages, including the sequences of 16 parasite lineages analyzed for the present study (Fig. 3). The trees were midpoint-rooted, resulting in one clade containing all *Leucocytozoon* lineages and another clade containing all other haemosporidian parasite lineages. The *Leucocytozoon* clade included three highly-supported subclades, the first comprising the majority of known *Leucocytozoon* lineages, the second including the clade of the *L. toddi* group, which can be further divided into two subclades of phylogenetically related lineages (L2 and L3 clades) and a third clade comprising *Leucocytozoon neotropicalis* lPIRIE01, *Leucocytozoon grallariae* lGRSQU01, *Leucocytozoon pterotenuis* lGRARUF01, *Leucocytozoon sabrazesi* lGALLUS08 and the new lineage *Leucocytozoon* sp. lPANHAL01. In both the BI and ML trees, *Leucocytozoon* (*Akiba*) *caulleryi* did not cluster with the other *Leucocytozoon* lineages but was part of the second main clade comprising other haemosporidian parasites. The deeper nodes of the second main clade (including *Plasmodium* and *Haemoproteus*) obtained low support values, not allowing precise conclusions to be drawn about the relationships between the subclades. In both the BI and ML, *Haemoproteus* (*Haemoproteus*) was the sister group to the clade containing the other taxa, but the position of *L.* (*Akiba*) *caulleryi* differed. In the BI tree, *L.* (*Akiba*) *caulleryi* was the second-most basal clade, but in the ML tree, it formed a trichotomy with *Plasmodium* and *Haemoproteus* (*Parahaemoproteus*), clustering with the *H. nisi* clade. In both trees, *Haemoproteus catharti* hCATAUR01 was the sister group to the clade containing the lineages of the family Plasmodiidae and the genus *Haemocystidium*. *Haemocystidium* is currently also classified into the family Haemoproteidae but is phylogenetically more closely related to the family Plasmodiidae. Within the Plasmodiidae clade, *Plasmodium* lineages from ungulates, birds and squamates constitute the more basal branches from which the other *Plasmodium* lineages diverge. The genus *Nycteria* forms the sister group to the clades of the *Plasmodium* subgenera *Paraplasmodium*, *Plasmodium*, *Laverania*, *Vinckeia* and *Hepatocystis*. *Nycteria* is embedded within the genus *Plasmodium*, although it is currently classified into a separate genus.

In addition, we calculated BI and ML trees based on an alignment that also included non-coding sequence regions (Additional file 5: Figure S1). The topology of these trees partially differed from that of the trees calculated solely with the three protein-coding genes (*cox1*, *cox3* and *cytb*) . In particular, the *H. nisi* H3 clade did not cluster with *Haemoproteus* (*Parahaemoproteus*) but formed the sister group to the clade comprising the other taxa (excluding *Leucocytozoon*), and *Haemoproteus* (*Haemoproteus*) was the second-most basal clade. Moreover, *L.* (*Akiba*) *caulleryi* clustered with *Haemoproteus* (*Parahaemoproteus*) with low support. Another difference was that *Plasmodium* (*Laverania*) did not form a reciprocally monophyletic clade with *Plasmodium* (*Vinckeia*) but was the sister group to a clade including *Plasmodium* (*Vinckeia*), *Plasmodium* (*Hepatocystis*) and *Plasmodium* (*Plasmodium*). The topology of the *Leucocytozoon* clade also changed considerably because the new lineage *Leucocytozoon* sp. lPANHAL01 was placed as the sister group to a clade containing all *Leucocytozoon* lineages except for the *L. toddi* group clade. By contrast, in the trees based on the three protein-coding genes, *Leucocytozoon* sp. lPANHAL01 formed a reciprocally monophyletic clade with *L. sabrazesi* lGALLUS08, *L. neotropicalis* lPIERIE01*, L. grallariae* lGRSQU01 and *L. pterotenuis* lGRARUF01, which again was the sister clade to the two *L. toddi* group clades (Fig. 3).

### Phylogeny of *L. toddi* group lineages

**Fig. 4:**
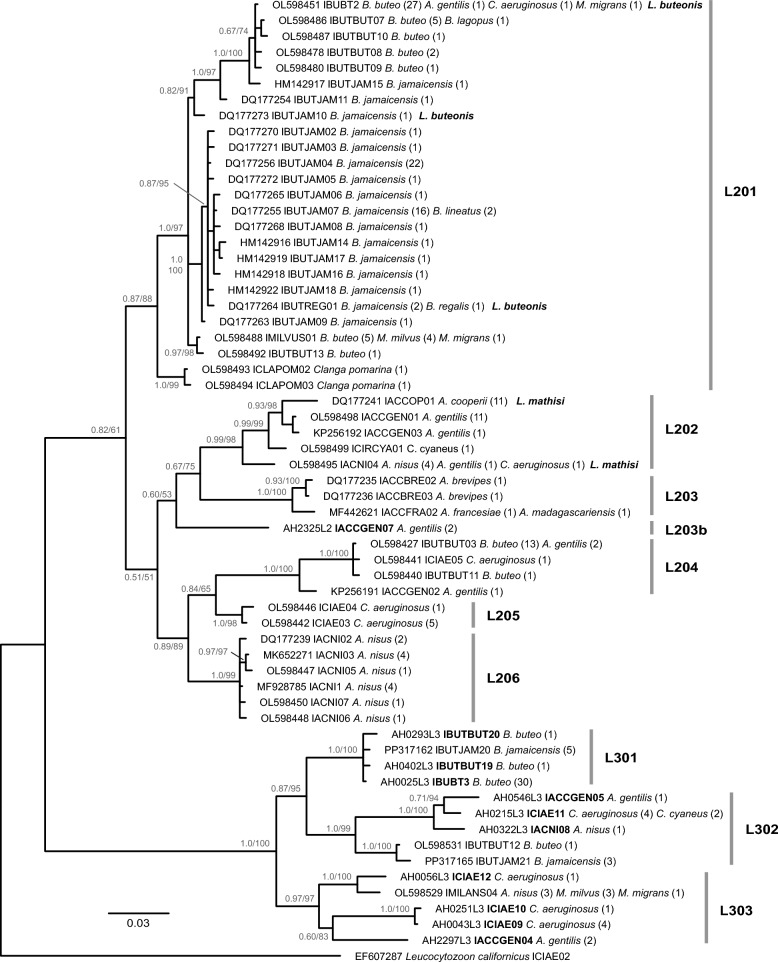
Phylogenetic relationships between parasite lineages of the *Leucocytozoon toddi* group based on partial (478 bp) *cytb* sequences. Bayesian Inference posterior probabilities and maximum likelihood bootstrap values are indicated at most nodes. The host species and number of individual records are provided for each lineage. Lineages first detected in the present study are marked in bold letters. The species names are indicated next to the lineages linked to the two morphospecies *Leucocytozoon buteonis* and *Leucocytozoon mathisi*

The *L. toddi* group tree was calculated with partial *cytb* sequences (478 bp) of 60 different lineages, 11 of which were first detected in the present study (Fig. 4). Ten of these lineages were obtained using the new nested primer set specifically targeting the *L. toddi* L3 clade. We roughly followed the division of subclades by Harl et al*.* [20], but renamed the subclades containing the lineages lMILANS04 and lBUTBUT12 (previously L207 and L208) to L302 and L303 in the phylogenetic tree. In the 478-bp *cytb* barcode sequence, the mean divergence (*p*-distance) between parasite lineages of the *L. toddi* L2 (L201–L206) and *L. toddi* L3 (L301–L303) clades was 16.7%, and the maximum *p*-distances between the lineages within the L2 and L3 clades were 12.8% and 12.1%, respectively.

## Discussion

The new nested-PCR approach reported here, which involves lineage-specific primers in the first PCR assays, successfully allowed the sequencing 18 complete mt genomes belonging to five *H. nisi* group and nine *L. toddi* group lineages, as well as two other *Leucocytozoon* lineages from samples containing mixed infections. We also detected 10 new parasite lineages of a previously neglected *L. toddi* clade (L3).

During the last decade, several PCR assays for sequencing the mt genomes of avian haemosporidian parasites have been published, all of which rely on high-quality DNA due to the large size of the amplified fragments. In most of these approaches, large parts of the mt genomes are amplified using long-range PCRs in a first step, followed by cloning and Sanger sequencing [3] or sequencing using Illumina or PacBio (Pacific Biosciences, Menlo Park, CA, USA) technology [18, 26]. The methods involving next-generation sequencing (NGS) technology are instrumental for analyzing large numbers of samples in parallel, and they enable sequencing of mt genomes even in samples with mixed infections. By contrast, the PCR assay reported by Musa [19], which relies on four separate nested PCRs and Sanger sequencing, is less suitable when the samples contain mixed infections. The nested PCR approach presented here also does not involve NGS technology but requires basic knowledge of primer design, unless the samples contain single infections. In this context, it is essential to note that mixed infections with multiple haemosporidian parasite lineages are common in birds [34, 35]. This is particularly the case for haemosporidian parasites of accipitriform raptors, most of which are not detected with the common *cytb* PCR assays [20, 21]. Moreover, a lineage might not be detected in *cytb* screenings if the parasitemia is lower than that of other lineages contained in the same samples or if the primers do not match equally well. Generally, it is crucial for all methods assembling mt genomes from overlapping PCR fragments that the electropherograms are carefully checked to ensure that the overlapping parts are identical and that the sequences do not contain ambiguities, which usually indicate the presence of mixed infections. Such close checking particularly important since the assembly of PCR fragments from different parasite lineages could lead to the creation of artificial mt genomes. The current approach of performing long-range PCRs with lineage- and group-specific primers prior to performing the nested PCRs helps to avoid this issue; however, the *cytb* sequences of all parasite lineages contained in the samples should be known a priori.

Parasites belonging to the *L. toddi* and *H. nisi* clades remained undetected in several earlier studies on haemosporidian parasites of accipitriform raptors. However, using alternative primer sets revealed the existence of numerous previously undetected parasite lineages [20, 21, 23, 24; present study].

The *L. toddi* group currently includes 60 parasite lineages isolated from 16 species of accipitriform raptors in Austria [20; present study], Czechia [36], Germany [37], Israel [38], Kazakhstan [23], Lithuania [23], Madagascar [39], Spain [25] and the USA [23, 24, 26]. Among the 171 accipitriform raptors from Austria, 31 *L. toddi* group lineages were detected, 10 of which were first discovered in the present study [21; present study]. Since the order Accipitriformes comprises about 260 species, most of which have not been included in molecular studies targeting haemosporidian parasites, this number likely represents only a fraction of the diversity of *L. toddi* group parasites. Moreover, the primers used to obtain partial *cytb* sequences of *L. toddi* group parasites in previous studies [20, 23, 36] have several mismatches relative to the *L. toddi* L3 clade, which either prevent or hinder amplification by PCR. Until now, the only published *L. toddi* L3 clade lineages were lMILANS04 [25], lBUTBUT12 [20], lBUTJAM20 and lBUTJAM21 [26], as well as an incomplete barcode sequence of lBUBT3 [37]. The nearly complete mt genomes of the lineages lBUTJAM20 and lBUTJAM21 (MalAvi database [2], updated 11/06/2025) were published only recently by Pacheco et al*.* [26], representing the first mt genomes of the *L. toddi* group. Although multiple morphologically similar *Leucocytozoon* species have been described from accipitriform raptors, Greiner and Kocan [40] synonymized most of them with *Leucocytozoon toddi* Sambon, 1908. Valkiūnas et al*.* [41] redescribed and genetically characterized *L. mathisi* (lACNI04: DQ177252, lACCOP01: DQ177250) and *L. buteonis* (lBUTJAM10: DQ177273, lBUBT2: DQ177253, lBUTREG01: DQ177264), but *L. toddi* is still not genetically characterized because molecular screenings performed to date have not included the type host *Kaupifalco monogrammicus*. However, based on the known genetic diversity and host specificity of the *cytb* lineages, the *L. toddi* group likely comprises numerous yet undescribed and genetically uncharacterized species.

Parasites of the *H. nisi* group were also common in the 171 accipitriform raptors from Austria, but the diversity was much lower, with only six lineages (hACCNIS06, hACCNIS07, hACCNIS08, hACCNIS09, hBUBT1 and hCIAE08). These are to date the only *H. nisi* group lineages for which the complete 478-bp *cytb* barcode sequences are available. The lineages hACCNIS06, hBUBT1 and hCIAE08 were also found in accipitriform raptors from France [21], and Svobodová et al*.* [42] published partial *cytb* barcode sequences (424 bp) of the lineages hACCNIS06 (ON375836) and hACCNIS07 (ON375837) from *Accipiter nisus* in Czechia. Ishak et al*.* [43] and Outlaw and Ricklefs [44] identified *H. nisi* group parasites in *B. jamaicensis* and *Accipiter cooperi* from the USA, but the published sequences only cover parts of the 478-bp barcode sequence; based on the available sequence parts downstream of the *cytb* barcode region, these sequences belong to at least eight different haplotypes. Four haplotypes isolated from *B. jamaicensis* resemble hBUBT1 (GQ141607, GQ141611, GQ141613, GQ141615, GQ141616, GQ141617 and FJ966927), two found in *A. cooperi* are similar to hACCNIS06 and hCIAE08 (FJ966921 and FJ966922) and another two from *A. cooperi* (FJ966920 and FJ966923) differ by several base pairs from the known *H. nisi* group lineages. Currently, *Haemoproteus nisi* Peirce and Marquiss 1983 (linked to hACCNIS06 and hCIAE08) and *Haemoproteus multivacuolatus* Harl et al*.* 2024 (linked to hBUBT1) are the only genetically characterized species of the *H. nisi* group. *Haemoproteus elani* Mello 1935 (type host *Elanus caeruleus*), *Haemoproteus janovyi* Greiner and Mundy 1979 (type host *Gyps africanus*) and *Haemoproteus buteonis* Wingstrand 1947 (type host *B. buteo*) likely belong to the *H. nisi* group as well but they have not been linked genetically [21]. *Haemoproteus multivacuolatus* shares the same type host with *H. buteonis*, but was described as a new species because the growing gametocytes induced an enlargement of infected erythrocytes, which was not the case in *H. buteonis* following the original description [21]. To clarify the taxonomic situation of *H. nisi* group parasites, it is crucial to investigate high-quality blood smears of a larger number of accipitriform raptors and to obtain the complete *cytb* barcode sequences of the lineages isolated from North American raptors.

The new *Leucocytozoon* lineage lPANHAL01 from an Austrian *Pandion haliaetus* is also of high interest because it is the first haemosporidian parasite lineage exclusively detected in ospreys, which is the most widely distributed species within the Accipitriformes. Lemus et al*.* [45] reported high prevalences of *L. toddi* (39.2%) and *Plasmodium polare* (13.7%) (determination uncertain) in osprey nestlings from Baja California (Mexico), but the study was not accompanied by molecular analyses. Pacheco et al*.* [26] isolated the lineages *P. elongatum* pGRW06 (PP317153) and *Plasmodium* sp. pMYCAME02 (PP317154) from ospreys sampled in the USA, both of which are parasite lineages found in numerous bird species. Other studies investigating ospreys from Europe [46, 47] and North America [40, 48, 49] did not detect haemosporidian parasites.

The phylogenetic relationships of haemosporidian parasites have been investigated in several studies using the three protein-coding mt genes (three protein-coding genes (*cox1*, *cox3* and *cytb*) [3, 4, 50], but the basal splits between the genera and subgenera are still not fully resolved. One of the main issues in solving phylogenetic relationships based on mt genomes is that evolutionary substitution rates differ strongly between the clades, even within the same genus [3]. For example, mt genomes of mammalian *Plasmodium* species have higher substitution rates than those of avian *Plasmodium* species, leading to longer branches in phylograms. Including sequences with different evolutionary rates can lead to the phenomenon of long-branch attraction in which longer branches tend to cluster with each other [51]. In this regard, the *H. nisi* clade has a much longer branch than the *Haemoproteus* (*Haemoproteus*) and *Haemoproteus* (*Parahaemoproteus*) clades, potentially affecting its position in the phylogenetic trees.

The phylogenetic analyses conducted in the present and earlier studies show that the genus *Leucocytozoon* is polyphyletic, as it does not include *Leucocytozoon* (*Akiba*) *caulleryi*, which takes a basal position in the clade containing the *Haemoproteus* and *Plasmodium* lineages. *Leucocytozoon* (*Akiba*) *caulleryi* is the only representative of the monotypic subgenus *Akiba* and is transmitted by biting midges (Ceratopogonidae), whereas the vectors of other *Leucocytozoon* parasites are black flies (Simuliidae) [3, 52]. Based on the currently available mt genome data, the genus *Leucocytozoon* could be subdivided into at least three subclades, with the first comprising most *Leucocytozoon* lineages from Passeriformes and other bird orders, the second including the *L. toddi* group lineages and the third containing *L. neotropicalis*, *L. grallariae*, *L. pterotenuis* and *L. sabrazesi*. The *L. toddi* group is unique because the parasites are exclusive to accipitriform raptors and primarily develop gametocytes in fusiform host cells [1, 41]. The latter feature was also described for parasites of the third subclade and approximately 15 molecularly uncharacterized *Leucocytozoon* species that primarily infect Galliformes and other non-passeriform birds [1, 53, 54], indicating that the development of gametocytes in fusiform host cells may be an ancestral trait of *Leucocytozoon* parasites. Based on their unique host distribution and strong divergence from other *Leucocytozoon* parasites, the *L. toddi* group could be classified as a subgenus.

The results of the phylogenetic analyses suggest that the genus *Haemoproteus* is polyphyletic. The *H. nisi* clade only clustered with the subgenus *Parahaemoproteus* in the tree based on the three protein-coding genes (*cox1*, *cox3* and *cytb*), but not when non-coding sequences were included, and the subgenus *Haemoproteus* did not cluster with the other two clades in any of the analyses. *Parahaemoproteus* is still considered to be a subgenus of *Haemoproteus* but had already been elevated to genus level by Bennett et al*.* [55], mainly because its parasites are transmitted by biting midges and those of the subgenus *Haemoproteus* by louse flies (Hippoboscidae) [55]. Moreover, parasites of the subgenus *Haemoproteus* are almost exclusively restricted to birds of the orders Columbiformes and Suliformes [56]. Earlier phylogenetic studies also did not recover the genus *Haemoproteus* as monophyletic, and the authors suggested elevating the subgenera to genus level [38, 57]. *Haemoproteus* parasites of accipitriform raptors, most of which likely belong to the *H. nisi* group, were previously not identified as a separate taxonomic group and are still considered to be part of the subgenus *Parahaemoproteus* [1, 56]*.* However, since these parasites are exclusive to accipitriform raptors and their mt genomes are strongly diverged from those of parasites of the subgenera *Haemoproteus* and *Parahaemoproteus*, a classification into a separate subgenus or even genus might be considered.

## Conclusions

In summary, by re-screening accipitriform raptor samples with a nested PCR approach targeting the previously neglected *L. toddi* clade, we uncovered 10 new parasite lineages, broadening the known diversity of these haemosporidians. Additionally, we established a robust nested PCR assay capable of amplifying and sequencing complete mt genomes of haemosporidian parasites, even from samples containing mixed infections. The application of long-range PCRs with lineage-specific primers placed inside the *cytb*, followed by nested PCRs with universal primers, provides an alternative to recently published NGS approaches. Using this method, we sequenced the complete mt genomes of five *H. nisi* group, nine *L. toddi* group and two other *Leucocytozoon* lineages. The mt genomes from parasites of these clades are important contributions to solving the phylogenetic relationships of clades within the Haemosporida. Moreover, the data are particularly valuable for future studies on haemosporidian parasites from accipitriform raptors, which still remain poorly investigated.

## Supplementary Information

**Additional file 1: Table S1.** Parasite lineages contained in accipitriform raptors from Austria.

**Additional file 2: Table S2. **Mitochondrial genomes and sequence partitions used in the phylogenetic analyses.

**Additional file 3: Tables S3–S8. **Nucleotide and amino acid compositions of the three mitochondrial protein-coding genes.

**Additional file 4: Text S1.** Nucleotide composition and codon usage of the three protein-coding mitochondrial genes.

**Additional file 5: Figure S1. **Phylogenetic relationships of haemosporidian parasites based on the three mitochondrial protein-coding genes and non-coding sequences.

## Data Availability

The sequences generated for the present study were uploaded to NCBI GenBank under the accession numbers PV872084–PV872130 ( *cytb*) and PV839571–PV839588 (mitochondrial genomes).
