# Supplementary material for: New mitochondrial genomes of parasites belonging to the Leucocytozoon toddi and Haemoproteus nisi groups (Haemosporida, Apicomplexa)
Source: Parasit Vectors. 2026 Jan 20;19:80. doi: 10.1186/s13071-026-07244-0 (PMC12903594; doi:10.1186/s13071-026-07244-0)
Supplement: Supplementary file 3 — Additional file 3: Tables S3–S8. Nucleotide and amino acid compositions of the three mitochondrial protein-coding genes. [file 13071_2026_7244_MOESM3_ESM.pdf]

Table S3: nucleotide composition *cox1*

	T(U)	C	A	G	G/C	A/T
***Leucocytozoon toddi* group L2**						
AH0236 *Leucocytozoon* sp. lBUTBUT03	39.1	16.2	28.4	16.3	32.5	67.5
AH0215 *Leucocytozoon* sp. lCIAE03	37.7	17.3	28.5	16.4	33.7	66.2
AH2325 *Leucocytozoon* sp. lACCGEN07	40.1	15.4	28.9	15.5	30.9	69.0
***Leucocytozoon toddi* group L3**						
AH0056 *Leucocytozoon* sp. lCIAE12	41.9	12.5	30.0	15.5	28.0	71.9
AH2297 *Leucocytozoon* sp. lACCGEN04	41.5	12.9	29.8	15.8	28.7	71.3
AH0322 *Leucocytozoon* sp. lACNI08	40.2	14.0	30.0	15.8	29.8	70.2
AH0215 *Leucocytozoon* sp. lCIAE11	40.1	13.9	29.8	16.1	30.0	69.9
AH0216 *Leucocytozoon* sp. lCIAE11	40.1	13.9	29.8	16.1	30.0	69.9
AH0822 *Leucocytozoon* sp. lBUBT3	39.6	13.6	31.3	15.4	29.0	70.9
AH0249 *Leucocytozoon* sp. lCIAE09	39.8	12.6	31.4	16.2	28.8	71.2
**other *Leucocytozoon***						
AH2344 *Leucocytozoon* sp. lPANHAL01	40.7	14.8	29.3	15.2	30.0	70.0
AH0391 *Leucocytozoon* sp. lCOCCOC01	40.6	15.2	29.4	14.8	30.0	70.0
***Haemoproteus nisi* group H3**						
AH0841 *Haemoproteus nisi* hACCNIS06	43.7	11.6	30.6	14.0	25.6	74.3
AH0799 *Haemoproteus nisi* hACCNIS06	43.7	11.6	30.6	14.0	25.6	74.3
AH0248 *Haemoproteus nisi* hCIAE08	43.9	11.5	30.7	13.9	25.4	74.6
AH1660 *Haemoproteus multivacuolatus* hBUBT1	43.9	11.3	30.7	14.2	25.5	74.6
AH0167 *Haemoproteus* sp. hACCNIS07	44.0	11.5	30.2	14.3	25.8	74.2
AH2328 *Haemoproteus* sp. hACCNIS09	44.5	11.3	30.1	14.0	25.3	74.6
**Average**	**41.4**	**13.4**	**30.0**	**15.2**	**28.6**	**71.4**

***Leucocytozoon toddi* group L2**

	T(U)	C	A	G	G/C
	39.1	16.2	28.4	16.3	32.5
	37.7	17.3	28.5	16.4	33.7
	40.1	15.4	28.9	15.5	30.9
Avg.	**39.0**	**16.3**	**28.6**	**16.1**	**32.4**

***Leucocytozoon toddi* group L3**

	T(U)	C	A	G	G/C
	41.9	12.5	30.0	15.5	28.0
	41.5	12.9	29.8	15.8	28.7
	40.2	14.0	30.0	15.8	29.8
	40.1	13.9	29.8	16.1	30.0
	40.1	13.9	29.8	16.1	30.0
	39.6	13.6	31.3	15.4	29.0
	39.8	12.6	31.4	16.2	28.8
Avg.	**40.5**	**13.3**	**30.3**	**15.8**	**29.2**

other *Leucocytozoon*

	T(U)	C	A	G	G/C
	40.7	14.8	29.3	15.2	30.0
	40.6	15.2	29.4	14.8	30.0
Avg.	**40.7**	**15.0**	**29.4**	**15.0**	**30.0**

***Haemoproteus nisi* group**

	T(U)	C	A	G	G/C
	43.7	11.6	30.6	14.0	25.6
	43.7	11.6	30.6	14.0	25.6
	43.9	11.5	30.7	13.9	25.4
	43.9	11.3	30.7	14.2	25.5
	44.0	11.5	30.2	14.3	25.8
	44.5	11.3	30.1	14.0	25.3
Avg.	**44.0**	**11.5**	**30.5**	**14.1**	**25.5**

Table S4: nucleotide composition *cox3*

	T(U)	C	A	G	G/C	A/T
***Leucocytozoon toddi* group L2**						
AH0236 *Leucocytozoon* sp. lBUTBUT03	42.9	14.0	29.5	13.6	27.6	72.4
AH0215 *Leucocytozoon* sp. lCIAE03	42.9	13.9	29.5	13.7	27.6	72.4
AH2325 *Leucocytozoon* sp. lACCGEN07	43.2	13.7	30.8	12.3	26.0	74.0
***Leucocytozoon toddi* group L3**						
AH0056 *Leucocytozoon* sp. lCIAE12	44.4	10.8	33.2	11.6	22.4	77.6
AH2297 *Leucocytozoon* sp. lACCGEN04	45.1	9.9	34.1	10.9	20.8	79.2
AH0322 *Leucocytozoon* sp. lACNI08	45.1	10.1	33.1	11.7	21.8	78.2
AH0215 *Leucocytozoon* sp. lCIAE11	45.1	10.3	33.6	11.1	21.4	78.7
AH0216 *Leucocytozoon* sp. lCIAE11	45.1	10.3	33.6	11.1	21.4	78.7
AH0822 *Leucocytozoon* sp. lBUBT3	44.8	9.7	34.8	10.7	20.4	79.6
AH0249 *Leucocytozoon* sp. lCIAE09	44.7	10.5	35.1	9.7	20.2	79.8
**other *Leucocytozoon***						
AH2344 *Leucocytozoon* sp. lPANHAL01	40.5	15.3	32.1	12.0	27.3	72.6
AH0391 *Leucocytozoon* sp. lCOCCOC01	42.0	13.3	32.9	11.7	25.0	74.9
***Haemoproteus nisi* group H3**						
AH0841 *Haemoproteus nisi* hACCNIS06	46.5	10.5	32.0	11.0	21.5	78.5
AH0799 *Haemoproteus nisi* hACCNIS06	46.6	10.4	32.0	11.0	21.4	78.6
AH0248 *Haemoproteus nisi* hCIAE08	45.8	10.9	32.3	11.0	21.9	78.1
AH1660 *Haemoproteus multivacuolatus* hBUBT1	47.3	9.6	31.9	11.3	20.9	79.2
AH0167 *Haemoproteus* sp. hACCNIS07	46.6	10.2	32.1	11.0	21.2	78.7
AH2328 *Haemoproteus* sp. hACCNIS09	46.3	10.0	32.4	11.3	21.3	78.7
**Average**	**44.7**	**11.3**	**32.5**	**11.5**	**22.8**	**77.2**

***Leucocytozoon toddi* L2 group**

	T(U)	C	A	G	G/C
	42.9	14.0	29.5	13.6	27.6
	42.9	13.9	29.5	13.7	27.6
	43.2	13.7	30.8	12.3	26.0
Avg.	**43.0**	**13.9**	**29.9**	**13.2**	**27.1**

***Leucocytozoon toddi* L3 group**

	T(U)	C	A	G	G/C
	44.4	10.8	33.2	11.6	22.4
	45.1	9.9	34.1	10.9	20.8
	45.1	10.1	33.1	11.7	21.8
	45.1	10.3	33.6	11.1	21.4
	45.1	10.3	33.6	11.1	21.4
	44.8	9.7	34.8	10.7	20.4
	44.7	10.5	35.1	9.7	20.2
Avg.	**44.9**	**10.2**	**33.9**	**11.0**	**21.2**

other *Leucocytozoon*

	T(U)	C	A	G	G/C
	40.5	15.3	32.1	12.0	27.3
	42.0	13.3	32.9	11.7	25.0
Avg.	**41.3**	**14.3**	**32.5**	**11.9**	**26.2**

***Haemoproteus nisi* group**

	T(U)	C	A	G	G/C
	46.5	10.5	32.0	11.0	21.5
	46.6	10.4	32.0	11.0	21.4
	45.8	10.9	32.3	11.0	21.9
	47.3	9.6	31.9	11.3	20.9
	46.6	10.2	32.1	11.0	21.2
	46.3	10.0	32.4	11.3	21.3
Avg.	**46.5**	**10.3**	**32.1**	**11.1**	**21.4**

Table S5: nucleotide composition *cytb*

	T(U)	C	A	G	G/C	A/T
***Leucocytozoon toddi* group L2**						
AH0236 *Leucocytozoon* sp. lBUTBUT03	39.1	15.6	31.2	14.1	29.7	70.3
AH0215 *Leucocytozoon* sp. lCIAE03	40.1	15.2	31.1	13.5	28.7	71.2
AH2325 *Leucocytozoon* sp. lACCGEN07	40.1	15.2	31.2	13.4	28.6	71.3
***Leucocytozoon toddi* group L3**						
AH0056 *Leucocytozoon* sp. lCIAE12	40.5	13.4	32.3	13.9	27.3	72.8
AH2297 *Leucocytozoon* sp. lACCGEN04	40.9	13.7	32.1	13.3	27.0	73.0
AH0322 *Leucocytozoon* sp. lACNI08	40.9	13.8	32.0	13.3	27.1	72.9
AH0215 *Leucocytozoon* sp. lCIAE11	40.8	13.4	32.3	13.4	26.8	73.1
AH0216 *Leucocytozoon* sp. lCIAE11	40.8	13.4	32.3	13.4	26.8	73.1
AH0822 *Leucocytozoon* sp. lBUBT3	41.1	13.4	31.7	13.9	27.3	72.8
AH0249 *Leucocytozoon* sp. lCIAE09	40.8	13.6	32.6	12.9	26.5	73.4
**other *Leucocytozoon***						
AH2344 *Leucocytozoon* sp. lPANHAL01	40.1	14.7	31.8	13.4	28.1	71.9
AH0391 *Leucocytozoon* sp. lCOCCOC01	40.8	14.9	31.0	13.3	28.2	71.8
***Haemoproteus nisi* group H3**						
AH0841 *Haemoproteus nisi* hACCNIS06	41.7	11.7	34.7	11.8	23.5	76.4
AH0799 *Haemoproteus nisi* hACCNIS06	41.7	11.7	34.7	11.8	23.5	76.4
AH0248 *Haemoproteus nisi* hCIAE08	42.3	11.5	34.3	11.9	23.4	76.6
AH1660 *Haemoproteus multivacuolatus* hBUBT1	42.0	11.5	34.4	12.1	23.6	76.4
AH0167 *Haemoproteus* sp. hACCNIS07	42.4	11.3	34.4	11.9	23.2	76.8
AH2328 *Haemoproteus* sp. hACCNIS09	42.8	11.3	34.6	11.3	22.6	77.4
**Average**	**41.1**	**13.3**	**32.7**	**12.9**	**26.2**	**73.8**

***Leucocytozoon toddi* L2 group**

	T(U)	C	A	G	G/C
	39.1	15.6	31.2	14.1	29.7
	40.1	15.2	31.1	13.5	28.7
	40.1	15.2	31.2	13.4	28.6
Avg.	**39.8**	**15.3**	**31.2**	**13.7**	**29.0**

***Leucocytozoon toddi* L3 group**

	T(U)	C	A	G	G/C
	40.5	13.4	32.3	13.9	27.3
	40.9	13.7	32.1	13.3	27.0
	40.9	13.8	32.0	13.3	27.1
	40.8	13.4	32.3	13.4	26.8
	40.8	13.4	32.3	13.4	26.8
	41.1	13.4	31.7	13.9	27.3
	40.8	13.6	32.6	12.9	26.5
Avg.	**40.8**	**13.5**	**32.2**	**13.4**	**27.0**

other *Leucocytozoon*

	T(U)	C	A	G	G/C
	40.1	14.7	31.8	13.4	28.1
	40.8	14.9	31.0	13.3	28.2
Avg.	**40.5**	**14.8**	**31.4**	**13.4**	**28.2**

***Haemoproteus nisi* group**

	T(U)	C	A	G	G/C
	41.7	11.7	34.7	11.8	23.5
	41.7	11.7	34.7	11.8	23.5
	42.3	11.5	34.3	11.9	23.4
	42.0	11.5	34.4	12.1	23.6
	42.4	11.3	34.4	11.9	23.2
	42.8	11.3	34.6	11.3	22.6
Avg.	**42.2**	**11.5**	**34.5**	**11.8**	**23.3**

Table S6: amino acid composition *cox1*

	Ala	Cys	Asp	Glu	Phe	Gly	His	Ile	Lys	Leu	Met	Asn	Pro	Gln	Arg	Ser	Thr	Val	Trp	Tyr
***Leucocytozoon toddi* group L2**																				
AH0236 *Leucocytozoon* sp. lBUTBUT03	6.1	1.7	2.1	1.5	8.8	8.6	3.4	9.6	1.1	14.3	3.8	4.2	4.0	0.8	2.1	6.7	8.0	7.6	1.7	4.2
AH0215 *Leucocytozoon* sp. lCIAE03	6.1	1.7	2.3	1.3	9.0	8.8	3.4	9.2	0.8	14.3	3.6	4.2	4.0	0.8	2.3	6.5	8.0	8.0	1.7	4.0
AH2325 *Leucocytozoon* sp. lACCGEN07	5.5	1.7	2.1	1.5	9.2	8.6	3.2	9.7	1.1	14.5	3.6	4.2	4.0	1.1	2.1	6.9	8.2	6.9	1.7	4.4
***Leucocytozoon toddi* group L3**																				
AH0056 *Leucocytozoon* sp. lCIAE12	4.6	1.9	2.5	1.3	8.4	8.6	3.2	11.8	1.3	14.3	3.6	4.0	3.6	1.1	1.9	7.8	7.6	6.7	1.7	4.4
AH2297 *Leucocytozoon* sp. lACCGEN04	4.2	1.9	2.5	1.3	9.2	8.8	3.2	10.9	1.3	14.5	3.2	4.0	3.6	1.1	1.9	8.2	7.6	6.7	1.7	4.4
AH0322 *Leucocytozoon* sp. lACNI08	4.2	1.9	2.5	1.3	8.4	8.8	3.2	12.2	1.3	14.7	3.4	4.0	3.6	1.1	1.9	8.0	7.6	6.1	1.7	4.4
AH0215 *Leucocytozoon* sp. lCIAE11	4.2	1.9	2.5	1.3	8.4	8.8	3.2	11.8	1.3	14.7	3.4	4.0	3.6	1.1	1.9	7.8	7.8	6.5	1.7	4.4
AH0216 *Leucocytozoon* sp. lCIAE11	4.2	1.9	2.5	1.3	8.4	8.8	3.2	11.8	1.3	14.7	3.4	4.0	3.6	1.1	1.9	7.8	7.8	6.5	1.7	4.4
AH0822 *Leucocytozoon* sp. lBUBT3	3.8	1.9	2.5	1.3	8.2	8.8	3.2	13.0	1.3	14.3	3.4	4.0	3.6	1.1	1.9	8.4	7.4	6.1	1.7	4.4
AH0249 *Leucocytozoon* sp. lCIAE09	3.8	1.9	2.5	1.3	8.4	8.8	3.4	10.5	0.8	14.1	3.4	4.4	3.4	0.8	2.3	8.8	7.8	7.8	1.7	4.2
**other *Leucocytozoon***																				
AH2344 *Leucocytozoon* sp. lPANHAL01	5.7	1.7	2.1	1.5	9.2	8.4	3.2	10.7	1.3	15.1	3.2	4.4	3.8	1.1	1.9	7.8	6.9	6.7	1.7	3.8
AH0391 *Leucocytozoon* sp. lCOCCOC01	5.7	1.3	2.1	1.5	8.2	7.4	3.4	11.6	0.8	13.9	4.0	4.0	4.4	1.1	2.3	7.6	7.6	7.1	1.7	4.6
***Haemoproteus nisi* group H3**																				
AH0841 *Haemoproteus nisi* hACCNIS06	4.0	1.5	2.3	1.5	10.1	7.8	2.7	12.6	1.5	13.0	4.4	5.5	4.0	0.8	1.9	8.0	5.9	5.5	1.7	5.7
AH0799 *Haemoproteus nisi* hACCNIS06	4.0	1.5	2.3	1.5	10.1	7.8	2.7	12.6	1.5	13.0	4.4	5.5	4.0	0.8	1.9	8.0	5.9	5.5	1.7	5.7
AH0248 *Haemoproteus nisi* hCIAE08	4.0	1.5	2.3	1.5	9.9	7.8	2.7	13.2	1.5	13.2	4.4	5.5	4.0	0.8	1.9	8.0	5.9	4.8	1.7	5.7
AH1660 *Haemoproteus multivacuolatus* hBUBT1	4.2	1.5	2.3	1.5	9.9	7.8	2.7	13.2	1.5	13.0	4.4	5.2	4.0	0.8	1.9	8.4	5.5	4.8	1.7	5.9
AH0167 *Haemoproteus* sp. hACCNIS07	4.4	1.5	2.3	1.5	10.1	7.8	2.7	13.2	1.3	13.0	4.4	5.2	4.0	0.8	2.1	8.2	5.5	4.8	1.7	5.7
AH2328 *Haemoproteus* sp. hACCNIS09	3.8	1.7	2.3	1.5	9.9	7.8	2.7	12.8	1.5	13.0	4.4	5.2	4.0	0.8	1.9	8.0	6.3	5.0	1.7	5.9
**Average**	**4.6**	**1.7**	**2.3**	**1.4**	**9.1**	**8.3**	**3.1**	**11.7**	**1.2**	**14.0**	**3.8**	**4.5**	**3.8**	**0.9**	**2.0**	**7.8**	**7.0**	**6.3**	**1.7**	**4.8**

Table S7: amino acid composition *cox3*

	Ala	Cys	Asp	Glu	Phe	Gly	His	Ile	Lys	Leu	Met	Asn	Pro	Gln	Arg	Ser	Thr	Val	Trp	Tyr
***Leucocytozoon toddi* group L2**																				
AH0236 *Leucocytozoon* sp. lBUTBUT03	3.6	2.4	0.8	4.0	10.0	4.0	3.6	10.4	2.0	13.6	3.2	2.4	2.0	1.6	2.0	10.8	6.0	9.6	1.2	6.8
AH0215 *Leucocytozoon* sp. lCIAE03	5.6	2.0	0.8	4.0	9.6	4.0	3.2	10.8	2.0	14.4	3.2	2.8	2.0	1.6	1.6	10.4	6.4	7.6	1.2	6.8
AH2325 *Leucocytozoon* sp. lACCGEN07	4.0	2.0	0.8	3.6	8.8	4.4	3.6	11.6	1.6	14.8	2.8	2.4	2.0	2.0	1.6	10.4	7.6	7.6	1.2	7.2
***Leucocytozoon toddi* group L3**																				
AH0056 *Leucocytozoon* sp. lCIAE12	1.6	3.6	2.0	4.4	10.0	4.0	2.4	12.1	2.0	16.5	2.0	3.2	1.2	2.0	2.0	8.0	8.4	4.0	1.2	9.2
AH2297 *Leucocytozoon* sp. lACCGEN04	1.6	3.2	2.0	4.4	10.8	4.8	2.4	13.3	3.2	16.5	2.0	3.2	1.2	1.2	2.0	7.6	8.4	3.2	1.2	7.6
AH0322 *Leucocytozoon* sp. lACNI08	1.6	3.2	2.0	4.0	10.0	4.4	2.8	13.7	2.4	16.1	2.0	3.2	1.2	0.8	2.0	8.0	8.0	4.8	1.2	8.4
AH0215 *Leucocytozoon* sp. lCIAE11	1.6	3.2	1.6	4.8	10.0	4.0	2.8	14.1	2.4	16.5	2.0	3.2	1.2	0.8	2.0	8.0	7.6	4.4	1.2	8.4
AH0216 *Leucocytozoon* sp. lCIAE11	1.6	3.2	1.6	4.8	10.0	4.0	2.8	14.1	2.4	16.5	2.0	3.2	1.2	0.8	2.0	8.0	7.6	4.4	1.2	8.4
AH0822 *Leucocytozoon* sp. lBUBT3	0.8	2.4	1.2	4.4	9.2	4.0	3.2	15.3	2.4	15.7	2.4	2.8	1.2	0.4	2.0	10.0	7.2	4.4	1.2	9.6
AH0249 *Leucocytozoon* sp. lCIAE09	1.6	3.2	2.0	3.6	9.6	3.2	2.4	12.9	3.2	17.7	2.0	3.2	0.8	2.0	1.2	8.8	9.2	4.0	1.2	8.0
**other *Leucocytozoon***																				
AH2344 *Leucocytozoon* sp. lPANHAL01	2.8	2.8	1.2	3.2	10.4	4.8	2.4	13.7	2.4	14.1	2.4	3.6	1.6	1.2	1.6	9.2	7.6	6.8	1.2	6.8
AH0391 *Leucocytozoon* sp. lCOCCOC01	3.2	2.4	1.6	3.2	10.8	3.6	2.8	11.2	2.0	12.1	3.6	2.4	1.2	2.4	2.0	11.7	7.2	8.0	1.2	7.2
***Haemoproteus nisi* group H3**																				
AH0841 *Haemoproteus nisi* hACCNIS06	2.4	2.8	1.2	5.2	10.0	2.8	3.2	15.6	1.2	12.8	1.6	2.4	1.6	2.0	1.6	11.6	6.0	6.0	1.6	8.4
AH0799 *Haemoproteus nisi* hACCNIS06	2.4	2.8	1.2	5.2	10.0	2.8	3.2	15.6	1.2	12.8	1.6	2.4	1.6	2.0	1.6	11.6	6.0	6.0	1.6	8.4
AH0248 *Haemoproteus nisi* hCIAE08	2.4	2.8	1.2	5.2	10.0	2.8	3.2	16.0	1.2	12.4	1.6	2.4	1.6	2.0	1.6	11.6	6.0	6.0	1.6	8.4
AH1660 *Haemoproteus multivacuolatus* hBUBT1	2.4	2.8	1.2	5.2	9.6	2.8	3.2	15.6	1.2	12.4	1.6	2.8	1.6	2.0	1.6	12.0	5.2	6.4	1.6	8.8
AH0167 *Haemoproteus* sp. hACCNIS07	2.4	2.8	1.2	5.2	9.6	2.8	3.2	16.0	1.2	12.4	1.6	2.8	1.6	2.0	1.6	11.6	5.6	6.0	1.6	8.8
AH2328 *Haemoproteus* sp. hACCNIS09	2.4	2.8	1.2	5.2	9.2	2.8	3.2	15.6	1.2	12.4	1.6	2.4	1.6	2.0	1.6	11.6	6.0	6.8	1.6	8.8
**Average**	**2.5**	**2.8**	**1.4**	**4.4**	**9.9**	**3.7**	**3.0**	**13.7**	**2.0**	**14.4**	**2.2**	**2.8**	**1.5**	**1.6**	**1.8**	**10.1**	**7.0**	**5.9**	**1.3**	**8.1**

Table S8: amino acid composition *cytb*

	Ala	Cys	Asp	Glu	Phe	Gly	His	Ile	Lys	Leu	Met	Asn	Pro	Gln	Arg	Ser	Thr	Val	Trp	Tyr
***Leucocytozoon toddi* group L2**																				
AH0236 *Leucocytozoon* sp. lBUTBUT03	4.5	1.9	2.1	1.1	9.3	6.4	2.9	10.6	2.7	14.4	2.4	3.7	4.5	2.1	2.1	7.5	5.6	6.1	2.7	7.5
AH0215 *Leucocytozoon* sp. lCIAE03	4.8	1.9	2.1	0.8	9.8	5.9	3.2	11.7	2.4	15.2	1.3	3.2	4.3	2.7	2.4	7.2	5.3	6.7	2.7	6.7
AH2325 *Leucocytozoon* sp. lACCGEN07	4.0	1.9	2.1	0.8	9.8	6.1	2.9	12.2	2.4	15.2	1.3	3.5	4.3	2.7	2.4	7.7	5.3	6.1	2.7	6.7
***Leucocytozoon toddi* group L3**																				
AH0056 *Leucocytozoon* sp. lCIAE12	5.1	2.9	1.9	1.1	9.3	5.6	2.4	12.5	2.4	14.1	2.1	3.7	4.5	2.4	2.1	6.4	5.6	5.9	2.4	7.7
AH2297 *Leucocytozoon* sp. lACCGEN04	5.1	2.9	1.9	1.1	9.3	5.6	2.4	12.0	2.4	14.4	2.1	3.7	4.3	2.4	2.1	7.5	5.6	5.6	2.4	7.5
AH0322 *Leucocytozoon* sp. lACNI08	4.8	3.2	1.9	1.1	8.8	5.6	2.4	13.3	2.1	14.1	2.4	3.7	4.5	2.7	2.4	6.6	5.3	5.0	2.4	8.0
AH0215 *Leucocytozoon* sp. lCIAE11	4.8	3.2	1.9	1.1	8.8	5.6	2.4	13.8	2.1	13.5	2.1	3.7	4.5	2.7	2.4	6.4	5.8	5.0	2.4	8.0
AH0216 *Leucocytozoon* sp. lCIAE11	4.8	3.2	1.9	1.1	8.8	5.6	2.4	13.8	2.1	13.5	2.1	3.7	4.5	2.7	2.4	6.4	5.8	5.0	2.4	8.0
AH0822 *Leucocytozoon* sp. lBUBT3	5.0	2.9	1.9	1.3	9.0	5.8	2.4	13.5	2.4	13.5	2.1	3.7	4.5	2.1	2.4	6.6	5.3	5.3	2.4	7.7
AH0249 *Leucocytozoon* sp. lCIAE09	5.0	2.9	1.9	1.1	9.0	5.6	2.7	13.5	2.9	14.3	2.1	3.7	4.5	2.1	1.9	6.6	5.6	4.2	2.4	8.0
**other *Leucocytozoon***																				
AH2344 *Leucocytozoon* sp. lPANHAL01	5.6	2.1	1.9	1.3	8.5	5.6	3.2	13.3	1.9	13.0	1.3	5.1	4.5	2.9	2.1	7.2	4.3	6.7	2.4	7.2
AH0391 *Leucocytozoon* sp. lCOCCOC01	5.3	1.3	1.6	1.3	10.6	5.9	2.7	11.7	2.4	13.8	1.9	4.0	5.3	2.9	2.4	6.7	4.5	6.1	2.4	7.2
***Haemoproteus nisi* group H3**																				
AH0841 *Haemoproteus nisi* hACCNIS06	3.2	1.3	1.9	1.1	9.3	5.3	2.4	16.0	3.2	12.8	2.1	5.3	4.8	2.7	2.1	7.7	4.3	4.5	2.7	7.5
AH0799 *Haemoproteus nisi* hACCNIS06	3.2	1.3	1.9	1.1	9.3	5.3	2.4	16.0	3.2	12.8	2.1	5.3	4.8	2.7	2.1	7.7	4.3	4.5	2.7	7.5
AH0248 *Haemoproteus nisi* hCIAE08	3.2	1.3	1.9	1.1	9.3	5.6	2.4	16.5	3.2	13.3	1.9	5.0	4.8	2.7	2.1	7.4	4.2	4.0	2.7	7.7
AH1660 *Haemoproteus multivacuolatus* hBUBT1	3.5	1.3	1.9	1.1	9.0	5.3	2.7	16.7	2.9	12.7	2.1	4.8	4.8	2.7	2.4	7.4	4.2	4.2	2.7	7.7
AH0167 *Haemoproteus* sp. hACCNIS07	3.5	1.3	1.9	1.1	9.3	5.3	2.7	17.5	3.2	11.7	2.1	4.8	4.8	2.7	2.4	7.4	4.2	4.0	2.7	7.7
AH2328 *Haemoproteus* sp. hACCNIS09	2.9	1.1	1.9	1.1	9.8	5.3	2.9	18.3	2.9	11.9	1.6	4.8	4.8	2.4	2.4	7.7	4.2	3.7	2.7	7.7
**Average**	**4.3**	**2.1**	**1.9**	**1.1**	**9.3**	**5.6**	**2.6**	**14.1**	**2.6**	**13.6**	**2.0**	**4.2**	**4.6**	**2.6**	**2.3**	**7.1**	**5.0**	**5.2**	**2.5**	**7.5**
