# Supplementary material for: New mitochondrial genomes of parasites belonging to the Leucocytozoon toddi and Haemoproteus nisi groups (Haemosporida, Apicomplexa)
Source: Parasit Vectors. 2026 Jan 20;19:80. doi: 10.1186/s13071-026-07244-0 (PMC12903594; doi:10.1186/s13071-026-07244-0)
Supplement: Supplementary file 4 — Additional file 4: Text S1. Nucleotide composition and codon usage of the three protein-coding mitochondrial genes. [file 13071_2026_7244_MOESM4_ESM.pdf]

Additional file 4

New mitochondrial genomes of parasites belonging to the *Leucocytozoon toddi* and *Haemoproteus nisi* groups (Haemosporida, Apicomplexa)

Josef Harl, Tanja Himmel, M. Andreína Pacheco, Herbert Weissenböck

Nucleotide composition and codon usage in the three coding genes of the new mitochondrial genomes

Cytochrome c oxidase subunit I

The mean G/C-content in the *cox1* gene was 32.4% (T: 39.0%, C: 16.3%, A: 28.6%, G: 16.1%) in the *L. toddi* L2 group, 29.2% (T: 40.5%, C: 13.3%, A: 30.3%, G: 15.8%) in the *L. toddi* L3 group, 30.0% (T: 40.7%, C: 15.0%, A: 29.4%, G: 15.0%) in the other two *Leucocytozoon* lineages, and 25.5% (T: 44.0%, C: 11.5%, A: 30.5%, G: 14.1%) in the *H. nisi* group. Although the G-content was similar in the *cox1* of all taxa, the C-content was particularly low in the *H. nisi* group. The start codons in the *cox1* gene were "ATT" for the new *Leucocytozoon* and "GTT" for the new *Haemoproteus* lineages. Since "GTT" is not a valid start codon according to the "mold, protozoan, and coelenterate" mitochondrial code, the following "ATT" probably serves as the start codon for the *H. nisi* group lineages. The stop codons were "TAA" and "TAT" for the *Leucocytozoon* lineages and "AAA" for the *Haemoproteus* lineages. The latter is not a valid stop codon, but other possible stop codons could not be identified downstream of the assumed end of the *cox1*.

Cytochrome c oxidase subunit III

The mean G/C-content in the *cox3* gene was 27.1% (T: 43.0%, C: 13.9%, A: 29.9%, G: 13.2%) in the *L. toddi* L2 group, 21.2% (T: 44.9%, C: 10.2%, A: 33.9%, G: 11.0%) in the *L. toddi* L3 group, 26.6% (T: 41.3%, C: 14.3%, A: 32.5%, G: 11.9%) in the other two *Leucocytozoon* lineages, and 21.4% (T: 46.5%, C: 10.3%, A: 32.1%, G: 11.1%) in the *H. nisi* group. Strikingly, the mean G/C-content was much higher in the *L. toddi* L2 clade (27.1%) compared to the *L. toddi* L3 clade (21.2%). The start codons in the *cox3* gene were "TTT" for the *L. toddi* group lineages and "TTC" for the other *Leucocytozoon* and *Haemoproteus* lineages. The latter codons are not valid according to the official code but are seemingly used as start codons by almost all haemosporidian parasites, including those of the *H. nisi* group. The stop codons were "TAA" for the new *H. nisi* and *L. toddi* L3 group lineages, and "CGG" for the *L. toddi L2* group lineages. The latter is not a valid stop codon, but other stop codons could not be identified downstream of the assumed end of the *cox3*.

Cytochrome b

The mean G/C-content in the *cytb* gene was 29.0% (T: 39.8%, C: 15.3%, A: 31.2%, G: 13.7%) in the *L. toddi* L2 group, 27.0% (T: 40.8%, C: 13.5%, A: 32.2%, G: 13.4%) in the *L. toddi* L3 group, 28.2% (T: 40.5%, C: 14.8%, A: 31.4%, G: 13.4%) in the other two *Leucocytozoon* lineages, and 23.3% (T: 42.2%, C: 11.5%, A: 34.5%, G: 11.8%) in the *H. nisi* group. Comparable to the *cox1* and *cox3* genes, the G/C-content was much lower in the *H. nisi* group than in the other taxa. The assumed start codons in the *cytb* gene were "ATG", "ACG", and "TTG" for the *L. toddi* group lineages and *Leucocytozoon* sp. lCOCCOC01, "GTG" for *Leucocytozoon* sp. lPANHAL01, and "AAC" and "AAT" for the *H. nisi* group lineages. Among these, "ACG", "AAC", and "AAT" are not valid start codons. Since all of these sequences include at least one "TTT" codon one to four codons upstream, the latter might serve as an alternative start codon for haemosporidian parasites. The stop codons were "TAA", "AAA", and "CAA" for the *L. toddi* group lineages and "TAA" for *Leucocytozoon* sp. lCOCCOC01 and *Leucocytozoon* sp. lPANHAL01. The codons "AAA" and "CAA" are not valid stop codons, but others could not be identified downstream. The *H. nisi* group *cytb* sequences were aberrant as they contained a unique 1 bp insert 39 bp from the assumed end of the *cytb*, leading to a "TAA" stop codon 8 bp downstream of the insert.
