# Supplementary material for: New mitochondrial genomes of parasites belonging to the Leucocytozoon toddi and Haemoproteus nisi groups (Haemosporida, Apicomplexa)
Source: Parasit Vectors. 2026 Jan 20;19:80. doi: 10.1186/s13071-026-07244-0 (PMC12903594; doi:10.1186/s13071-026-07244-0)
Supplement: Supplementary file 5 — Additional file 5: Figure S1. Phylogenetic relationships of haemosporidian parasites based on the three mitochondrial protein-coding genes and non-coding sequences. [file 13071_2026_7244_MOESM5_ESM.pdf]

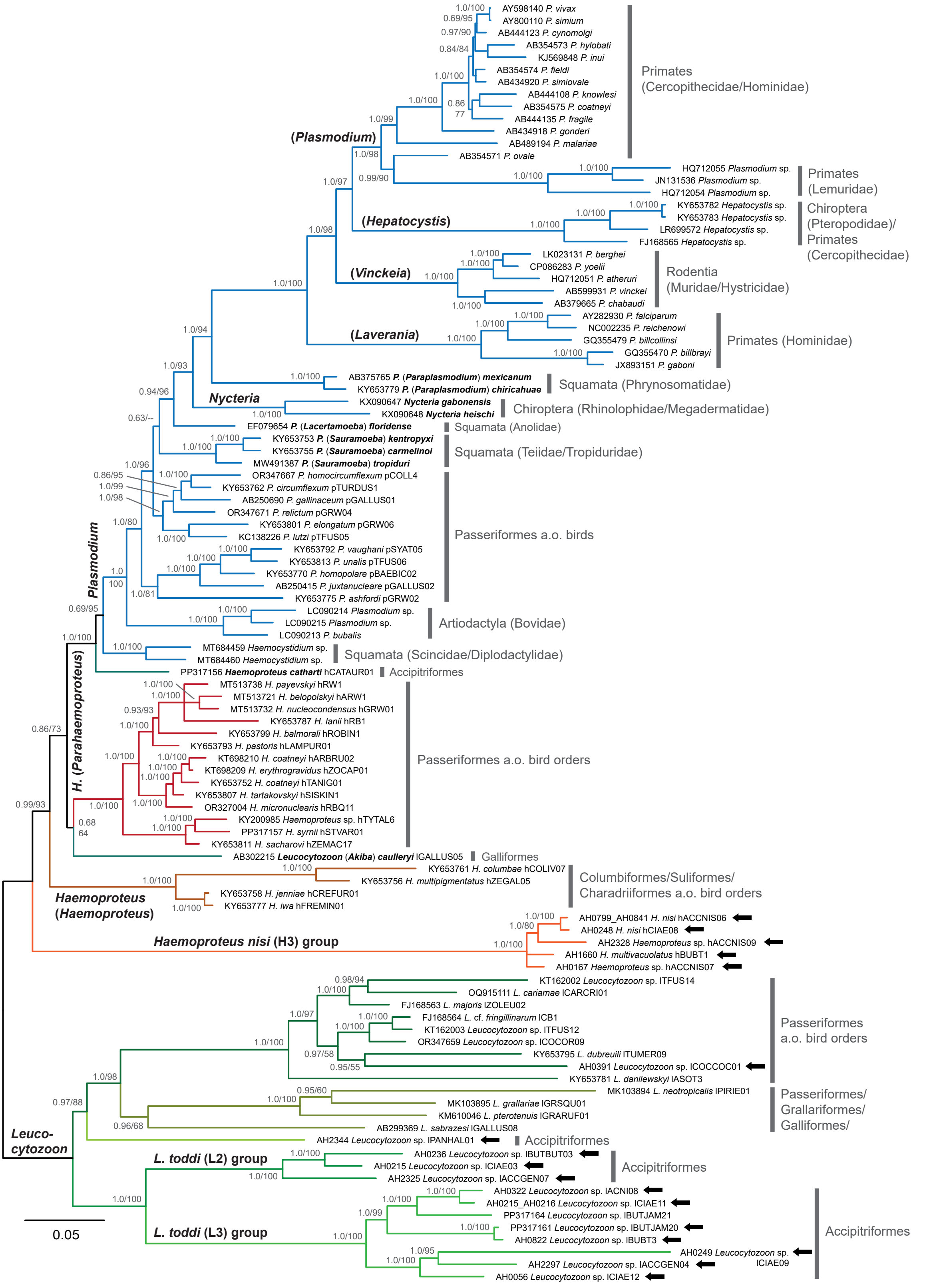

Additional File 5 Figure S1: Phylogenetic relationships between haemosporidian parasite lineages based on the concatenated sequences of the three mitochondrial protein-coding genes *COI* (1431 bp), *COIII* (753 bp), *CytB* (1131 bp), and non-protein coding sequences (2169 bp, gaps excluded). Bayesian Inference posterior probabilities and Maximum Likelihood bootstrap values are indicated at all nodes. Bold black arrows mark the lineages of which the mitochondrial genomes were sequenced for the present study. For haemosporidian parasites of bird hosts, the name of the MalAvi lineage is included in the label. The main host groups are indicated to the right of the clades, whereby "a.o." refers to "and other".
